# *Protein phosphatase 1 catalytic subunit gamma* is a causative gene for meat lightness and redness

**DOI:** 10.1371/journal.pgen.1011467

**Published:** 2024-11-20

**Authors:** Jiahong Sun, Xinting Yang, Guiping Zhao, Zhengxiao He, Wenhao Xing, Yanru Chen, Xiaodong Tan, Mengjie Wang, Wei Li, Bingxing An, Zhangyuan Pan, Zhengkui Zhou, Jie Wen, Ranran Liu

**Affiliations:** State Key Laboratory of Animal Biotech Breeding, Institute of Animal Sciences, Chinese Academy of Agricultural Sciences, Key Laboratory of Animal (Poultry) Genetics Breeding and Reproduction, Ministry of Agriculture and Rural Affairs, Beijing, China; Wageningen University & Research, NETHERLANDS, KINGDOM OF THE

## Abstract

The quality of meat is important to the consumer. Color is a primary indicator of meat quality and is characterized mainly into lightness, redness, and yellowness. Here, we used the genome-wide association study (GWAS) and gene-based association analysis with whole-genome resequencing of 230 fast-growing white-feathered chickens to map genes related to meat lightness and redness to a 6.24 kb QTL region (GGA15: 6298.34–6304.58 kb). This analysis revealed that only the *protein phosphatase 1 catalytic subunit gamma* (*PPP1CC*) was associated with meat color (*P* = 8.65E-08). The causal relationships between *PPP1CC* expression and meat lightness/redness were further validated through Mendelian randomization analyses (*P* < 2.9E-12). Inducible skeletal muscle-specific *PPP1CC* knockout (*PPP1CC*-SSKO) mice were generated and these mice showed increased lightness and decreased myoglobin content in the limb muscles. In addition, the predominant myofiber shifted from slow-twitch to fast-twitch myofibers. Through transcriptome and targeted metabolome evidence, we found that inhibition of *PPP1CC* decreased the expression of typical slow-twitch myofiber and myofiber-type specification genes and enhanced the glycolysis pathway. Functional validation through a plasmid reporter assay revealed that a SNP (rs315520807, C > T) located in the intron of *PPP1CC* could regulate the gene transcription activity. The differences in meat color phenotypes, myoglobin content, frequency of rs315520807 variant, expression of *PPP1CC* and fast-twitch fiber marker genes were detected between fast-growing white-feathered chickens and local chickens. In this study, *PPP1CC* was identified as the causative gene for meat color, and the novel target gene and variant that can aid in the innovation of meat improvement technology were detected.

## Author summary

Color is a primary indicator of the biochemical and physiological properties of meat and affects consumer perception. Meat color is characterized mainly into lightness, redness and yellowness. The causative gene of meat lightness and redness has not yet been identified. We found that the expression of *protein phosphatase 1 catalytic subunit gamma* (*PPP1CC*) was causally related to breast muscle lightness and redness in fast-growing white-feathered chickens using a GWAS, gene-based association analysis and Mendelian randomization analysis. Using mice with inducible, skeletal muscle-specific knockout of *PPP1CC* (*PPP1CC*-SSKO), we also revealed that lightness increased and myoglobin content decreased in the limb muscle and identified that myofiber-type specification is one of the mechanisms by which *PPP1CC* influences meat color. We subsequently discovered an intron variant (rs315520807, C > T) in *PPP1CC* that could regulate the gene transcription. The causative gene and potential causative variant for chicken lightness and redness could be utilized in the innovation of meat improvement technology.

## Introduction

Meat is one of the most important sources of high-quality proteins for humans. Meat quality is important to the consumer and represents a critical issue for the livestock production industry [1–4]. Meat quality traits are complex and are usually determined by various intrinsic and extrinsic factors [5]. Meat color is the first sensory information available to consumers and is determined by the dimensions of lightness, redness, and yellowness. Redness and yellowness are chromatically determined by pigment content, and lightness is chromatically determined by microstructure [6] Meat color not only influences appearance but is also correlated with pH, muscle fiber characteristics and lipid deposition. Severe myopathy related to meat color, including pale, soft, exudative (PSE) meat, results in consumer rejection and severe economic losses in the industry.

Understanding the genetic basis of meat color is important for the innovation of modern meat improvement technology. Some causative or related genes for meat color have been identified in chickens and domestic pigs. Strong relationships between beta-carotene oxygenase 1 (*BCO1*) [7] and chicken yellowness and between myosin heavy chain 3 (*MYH3*) [8] and redness/intramuscular fat in pig skeletal muscle have been reported. Changes in meat lightness might be related to changes in muscle glycolytic potential [9]. The Rendement Napole gene (*RN*) [10] and phosphorylase kinase catalytic subunit gamma 1 (*PHKG1*) [11] were found to be involved in glycolytic potential. However, the causal genes for meat lightness and redness have not been identified.

Efficient strategies for exploring functional genes include genome-wide association studies (GWASs) and gene-based association analysis [12–17]. Major QTLs and candidate genes affecting complex quantitative traits have been identified [15]; however, causal relationships are difficult to determine. In recent years, the Mendelian randomization (MR) method has been widely applied in the search for disease/pathogenic causal factors [18,19]. The MR method uses genetic variants to determine whether an observational association between a risk factor and an outcome trait is consistent with a causal effect [20].

Meat color is a complex trait because it is influenced by both muscle development and tissue metabolism. Muscle cells remain metabolically active after slaughter. Studying animals *in vivo* is the preferred approach for verifying gene function and exploring the underlying mechanisms involved. However, this approach requires a time-inducible and skeletal muscle-specific knockout mouse model with a high degree of mimicry, in which normal gene expression during embryonic and early development ensures normal tissue development. The knockout of a candidate gene that has broad roles and is functionally important [21] at an early age will result in severe harm. A relatively short induction period to downregulate target gene expression was more suitable for the study of post-slaughter traits.

In the present study, the causative gene and potential causative variant of chicken breast muscle color lightness and redness were identified through a combination of GWAS, gene-based association analysis, and MR analysis. Further experiments in inducible skeletal muscle-specific knockout (*SSKO*) mice verified the gene function, and the underlying regulatory mechanisms were revealed through targeted metabolome and transcriptome analyses.

## Results

### Identification of *Protein phosphatase 1 catalytic subunit gamma* (*PPP1CC*) as a putative quantitative trait gene (QTG) for chicken meat color lightness(L*) and redness(a*)

The descriptive statistics of the meat color traits of 230 fast-growing white-feathered chickens are shown in Table 1. The mean value for meat lightness (L*15 min) was 52.43, that for redness (a*15 min) was 10.25, and that for yellowness (b*15 min) was 12.77. The coefficient of variation of L*15 min was 3.48%, that of a*15 min was 14.60%, and that of b*15 min was 15.91%.

**Table 1:** Descriptive statistics of meat color in fast-growing white-feathered chickens.

Traits	N	Min	Max	Mean	SD	CV (%)
L*15 min	230	47.70	57.33	52.43	1.82	3.48
a*15 min	230	2.08	16.19	10.25	1.50	14.60
b*15 min	230	6.08	18.92	12.77	2.03	15.91

**Fig 1:**
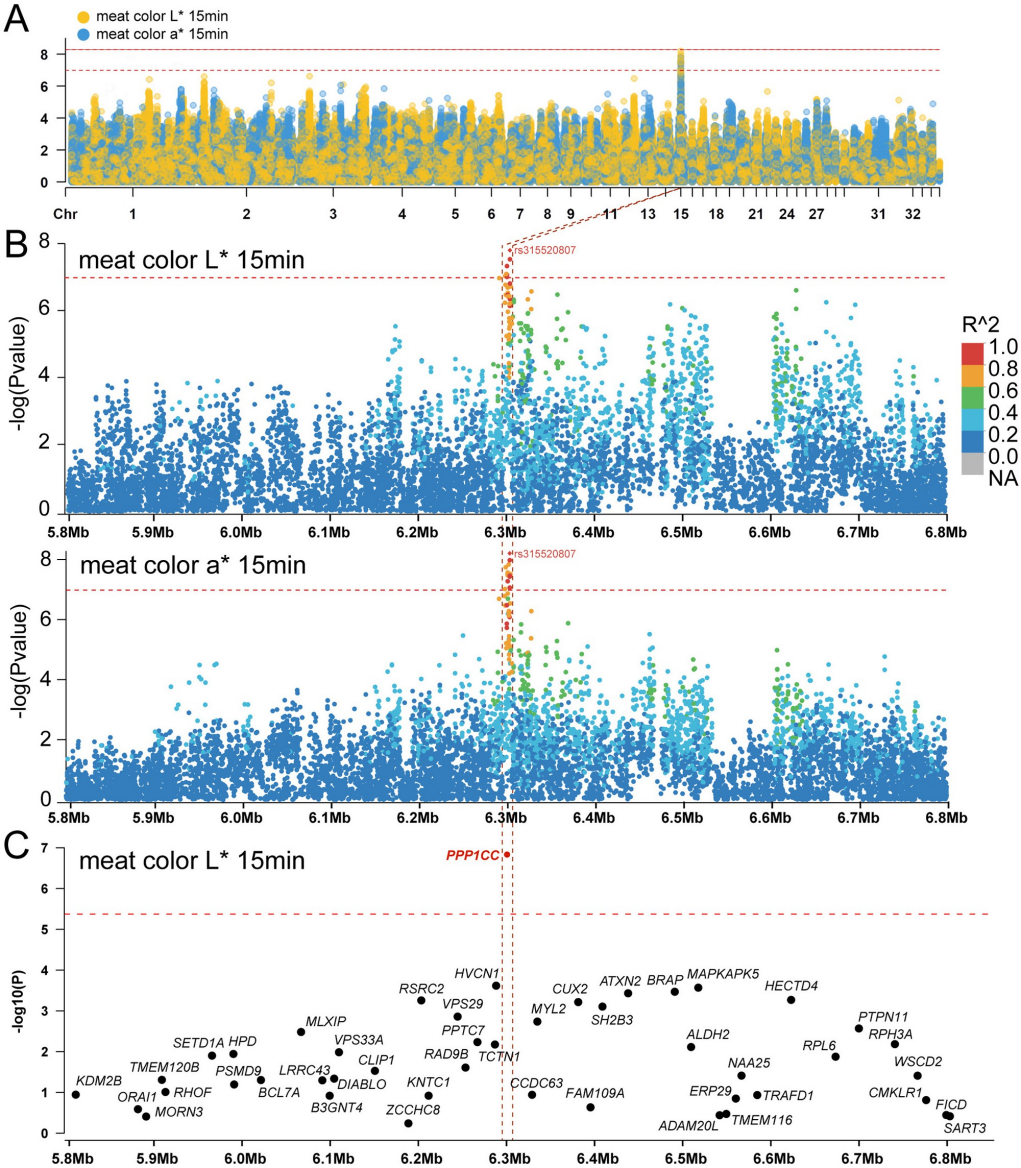
Identification of quantitative trait gene (QTG) for meat color lightness (L*) and redness (a*) in chickens. (A) Manhattan plot showing the genetic effects on meat color L*15 min and a*15 min according to a GWAS in 230 fast-growing white-feathered chickens. The solid and dashed lines indicate the whole-genome and suggestive significance thresholds, respectively. The yellow dots indicate SNPs associated with L*15 min, and blue dots indicate SNPs associated with a*15 min. (B) Regional plots for the loci GGA15: 5.80–6.80 Mb associated with meat color L*15 min and a*15 min. The level of linkage disequilibrium (LD) between the lead SNP rs315520807 and surrounding SNPs is indicated by r^2^. The lead SNP rs315520807 is highlighted by a red diamond. The 6.24 kb region (GGA15: 6298.34–6304.58 kb) with r^2^ > 0.8 is indicated by red dashed lines. (C) Gene-based association analysis using GWAS summary statistics for the meat color L*15 min by MAGMA. The region showed is from GGA15:5.80–6.80 Mb. The solid line indicates the whole-genome significance threshold (*P* < 0.05/11,821 = 4.23E-06).

To identify QTLs affecting chicken meat color, we performed a GWAS for the L* and a* values of breast muscle from 230 fast-growing white-feathered chickens with 9,760,228 single-nucleotide polymorphisms (SNPs). A single association peak was observed on chromosome 15 (Figs 1A and S1). A total of eight SNPs for L*15 min and 14 SNPs for a*15 min reached the suggestive significance threshold (*P* = 1.02E-07); these SNPs spanned a QTL interval from 6298.34 to 6304.58 kb along chromosome 15 (S1 Table). The details of the lead SNP rs315520807 (GGA15: 6298343) are shown in Table 2.

**Table 2:** Information of QTL interval and lead SNP significantly associated with meat color in fast-growing white-feathered chickens.

Traits	Chr.	Base-pair region		nSNP	Lead SNP	Alleles	MAF	*P* value
		Start	End		rsname			
L*15 min	15	6298343	6303822	8	rs315520807	C/T	0.31	1.59E-08
a*15 min	15	6298343	6304584	14	rs315520807	C/T	0.31	6.23E-09

We next calculated the pairwise linkage disequilibrium (LD) between the SNPs within 500 kb upstream and downstream of the primary locus and the lead SNP. Six significant SNPs spanning a region from 6298.34 kb to 6304.58 kb had strong LD (r^2^ > 0.8, Fig 1B).

Moreover, we performed gene-based association analysis and identified 11,821 gene–trait association pairs (Figs 1C and S2). The results revealed that only *PPP1CC* was significantly associated with the meat color L* and a* values (*P* = 8.65E-08 and 1.47E-07, respectively).

### Verifying a causal relationship between *PPP1CC* expression and meat color by Mendelian randomization analysis

**Fig 2:**
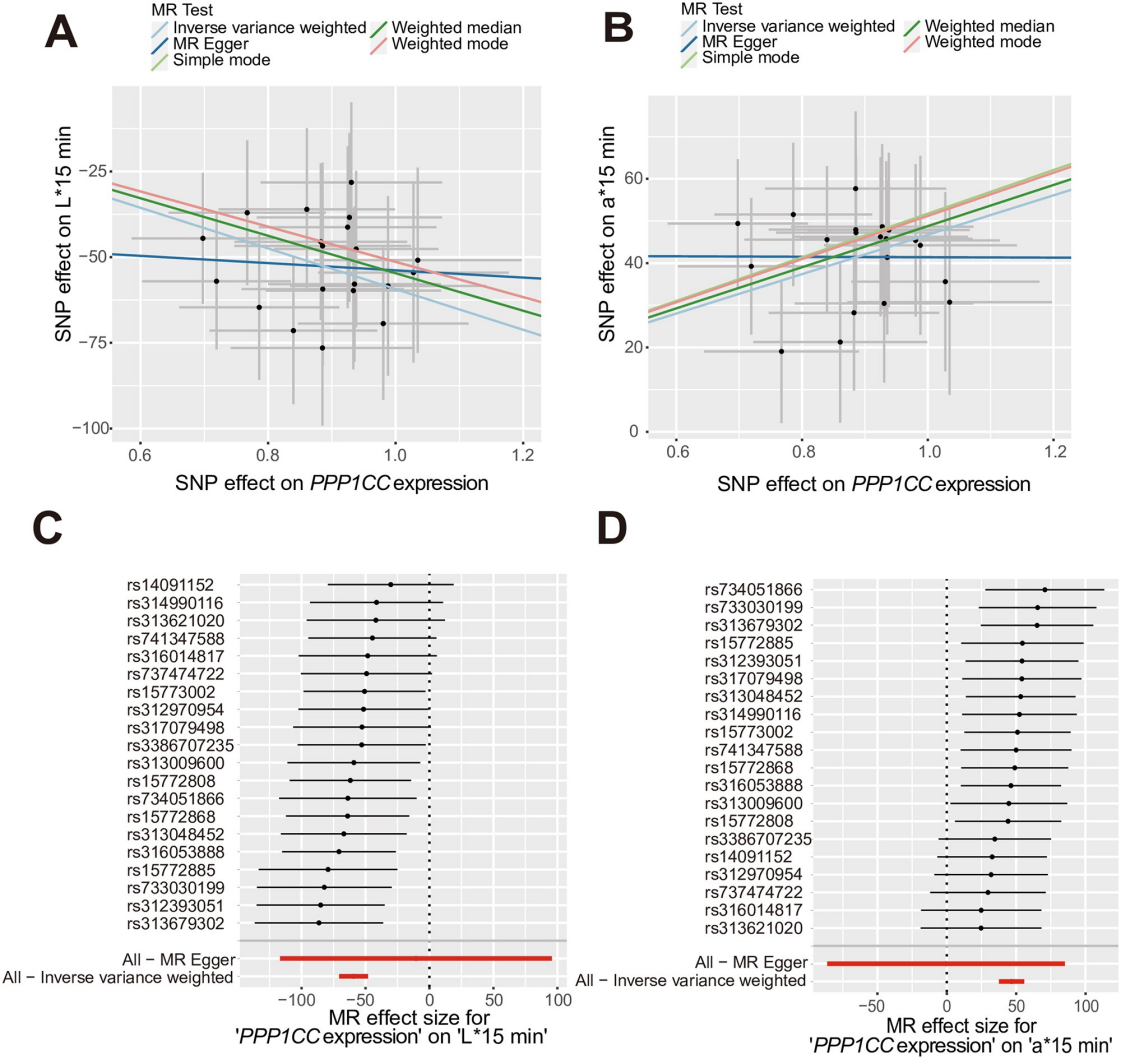
Mendelian randomization analysis results for *PPP1CC* expression in muscle L*15 min/a*15 min. (A) MR results for *PPP1CC* in L*15 min and (B) a*15 min based on different models. Each dot indicates the effect size of SNPs on related traits. The vertical error bars around each dot indicate the standard error of the estimated association between the SNP and L*15 min/a*15 min. The horizontal error bars indicate the standard error of the estimated association between the SNP and the expression of *PPP1CC*. The regression lines with different colors indicate the MR results generated by different models. (C) Forest plots showing individual SNP effects on L*15 min and (D) a*15 min. The last red line shows the effects of *PPP1CC* on L*15 min and a*15 min according to IVW methods.

The causal relationship between L*15 min and *PPP1CC* expression was robust (*P*_Weighted median_ < 2.91E-12, *P*_Inverse variance weighted_ < 8.32E-25). Additionally, a strong causal association was observed between a*15 min and *PPP1CC* expression (*P*_Weighted median_ < 1.16E-14, *P*_Inverse variance weighted_ < 8.54E-24) (S2 Table). The scatter diagram illustrates the negative effect of *PPP1CC* on L*15 min (Fig 2A) and the positive effect of *PPP1CC* on a*15 min (Fig 2B). However, none of the curves in the scatter diagram passed through the origin point. Consistent individual effects of *PPP1CC* SNPs on L*15 min and a*15 min are depicted in the forest plots (Fig 2C and 2D).

All causal associations showed no evidence of pleiotropy (*P* > 0.05, S3 Table). The leave-one-out sensitivity analysis revealed that the causal inference was not substantially driven by any individual SNP (S3A and S3B Fig). All instrumental variables (IVs) showed no evidence of heterogeneity (*Q* > 0.05, S3 Table). The funnel plot also revealed no evidence of obvious heterogeneity across the estimates, indicating the absence of potential pleiotropic effects (S3C and S3D Fig).

These results provide solid evidence that meat color traits (L*15 min and a*15 min) may be regulated by *PPP1CC* gene expression. The curves in the scatter diagram revealed that there were still some effects on meat color traits other than *PPP1CC* gene expression, but these effects were independent of these instrument variables (IVs).

### Inducible skeletal muscle-specific knockout of *PPP1CC* in mice led to changes in meat color and myofiber type

**Fig 3:**
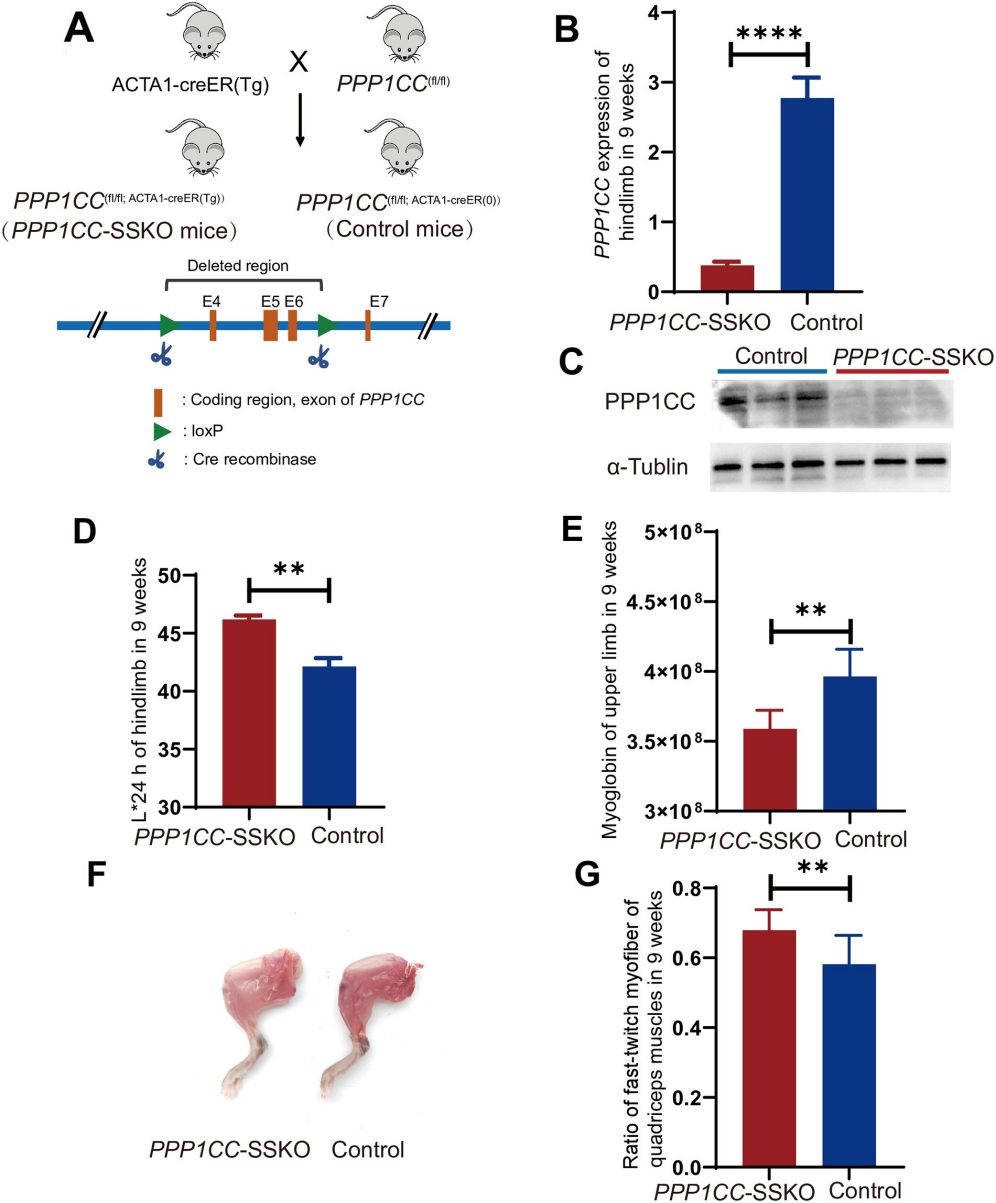
Changes in meat color and related indices in inducible skeletal muscle-specific *PPP1CC* knockout (*PPP1CC*-SSKO) mice. (A) Schematic diagram depicting the construction of *PPP1CC*-SSKO mice. (B) Relative *PPP1CC* mRNA expression in the hindlimb muscle of *PPP1CC*-SSKO and control mice (n = 10:10). (C) Western blot results of PPP1CC from the quadriceps muscle in *PPP1CC*-SSKO and control mice (n = 3:3). (D) L*24 h values of hindlimb muscles from *PPP1CC*-SSKO and control mice (n = 9:8). (E) Myoglobin content in the upper limbs of *PPP1CC*-SSKO and control mice (n = 10:10). (F) Pictures of the hindlimb muscles of *PPP1CC*-SSKO (left) and control (right) mice. (G) Ratio of fast-twitch myofibers in the quadriceps muscles of *PPP1CC*-SSKO and control mice (n = 4:3). *P < 0.05, **P < 0.01, ****P < 0.0001. The mouse cotton image was obtained from OpenClipart (https://openclipart.org/detail/17558/simple-cartoon-mouse). All phenotypes collected and samples tested were from 9-week-old mice that were injected with tamoxifen for 5 days at 6 weeks of age.

The *PPP1CC*-SSKO mice with a genotype of *PPP1CC* ^(fl/fl; ACTA1-creER(Tg))^ and control mice with a genotype of *PPP1CC* ^(fl/fl; ACTA1-creER(0))^ were constructed using the Cre/loxP system (Fig 3A). We injected tamoxifen at 6 weeks of age for 5 consecutive days to activate the ACTA1-Cre enzyme in skeletal muscle. The phenotypes and samples were collected at 9 weeks of age. *PPP1CC* mRNA expression in *PPP1CC*-SSKO mice was 13.35% of that of the control mice at 9 weeks of age (*P* < 0.0001) (Fig 3B). The PPP1CC protein levels were obviously decreased in *PPP1CC*-SSKO mice, as determined via Western blot (Fig 3C). These results indicated that the tamoxifen-induced knockdown of *PPP1CC* was successful. In terms of phenotype, the hindlimb muscle of *PPP1CC*-SSKO mice presented increased L*24 h (*P* < 0.01), and the upper limb muscle presented decreased myoglobin content (Fig 3D, 3E and 3F). NADH staining of muscle sections revealed that fast-twitch (type II) myofibers were more abundant (*P* < 0.01) in the quadriceps muscles of *PPP1CC*-SSKO mice, whereas the quadriceps muscles of the control group contained more slow-twitch (type I) fibers (Figs 3G and S4).

### Inducible skeletal muscle-specific knockout of *PPP1CC* in mice led to changes in myofiber-type specification and glycolysis

At 9 weeks of age, the transcriptomes of the hindlimb muscles were determined, revealing 50 significant Gene Ontology (GO) biological process terms related to muscle function among the differentially expressed genes (S4 Table). The GO terms were related mainly to muscle function, cytoskeleton, and myofiber activity (S5 Fig).

**Fig 4:**
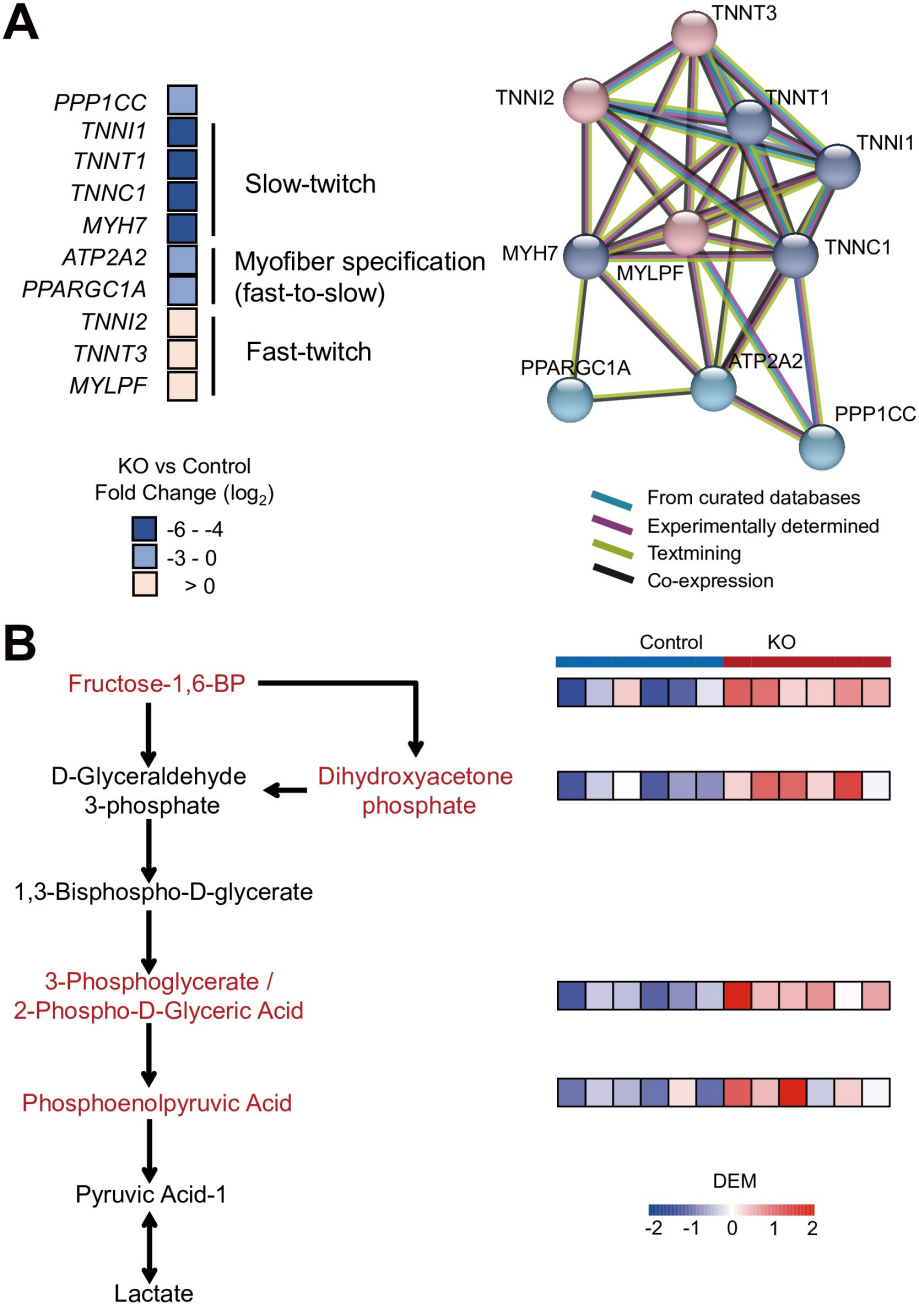
Transcriptome and targeted metabolome analyses of muscles from *PPP1CC*-SSKO and control mice. (A) Changes in the expression of genes related to slow-twitch myofibers, myofiber-type specification (fast-to-slow), and fast-twitch myofiber-related genes in *PPP1CC*-SSKO mice. The data are presented as log2 fold changes compared with those of the controls. Protein–protein interaction (PPI) network analysis was conducted for *PPP1CC* and myofiber-related genes. Each filled node denotes a gene; edges between nodes indicate PPIs between protein products of the corresponding genes. Different edge colors represent the types of evidence for the association. (B) Schematic representation of key metabolites related to glycolysis in *PPP1CC*-SSKO mice. Heatmap showing key metabolite changes between control and *PPP1CC*-SSKO and control mice.

The expression of slow-twitch myofiber marker genes (*TNNI1*, *TNNT1*, *TNNC1*, and *MYH7*) was significantly decreased, and the expression of fast-twitch marker genes (*TNNI2*, *TNNT3*, and *MYLPF*) tended to increase. With respect to the myofiber-type specification genes, the fast-to-slow myofiber-type specification genes *ATP2A2* and *PPARGC1A* decreased significantly (Fig 4A and S5 Table). The slow-twitch myofiber marker genes, fast-twitch marker genes, and *PPP1CC* were clustered into one network (Fig 4A).

Targeted metabolomics analysis revealed that the significantly differently abundant metabolites fructose-1,6-BP, dihydroxyacetone phosphate, 3-phosphoglycerate/2-phospho-d-glyceric acid, and phosphoenolpyruvic acid involved in glycolysis were highly upregulated in PPP1CC-SSKO mice (Fig 4B and S6 Table). Transcriptome-based analysis of differentially expressed genes also revealed a trend toward upregulation of genes in the glycolytic pathway (map00010) (S6 Fig).

### Functional analysis of the potential causative variant SNP

**Fig 5:**
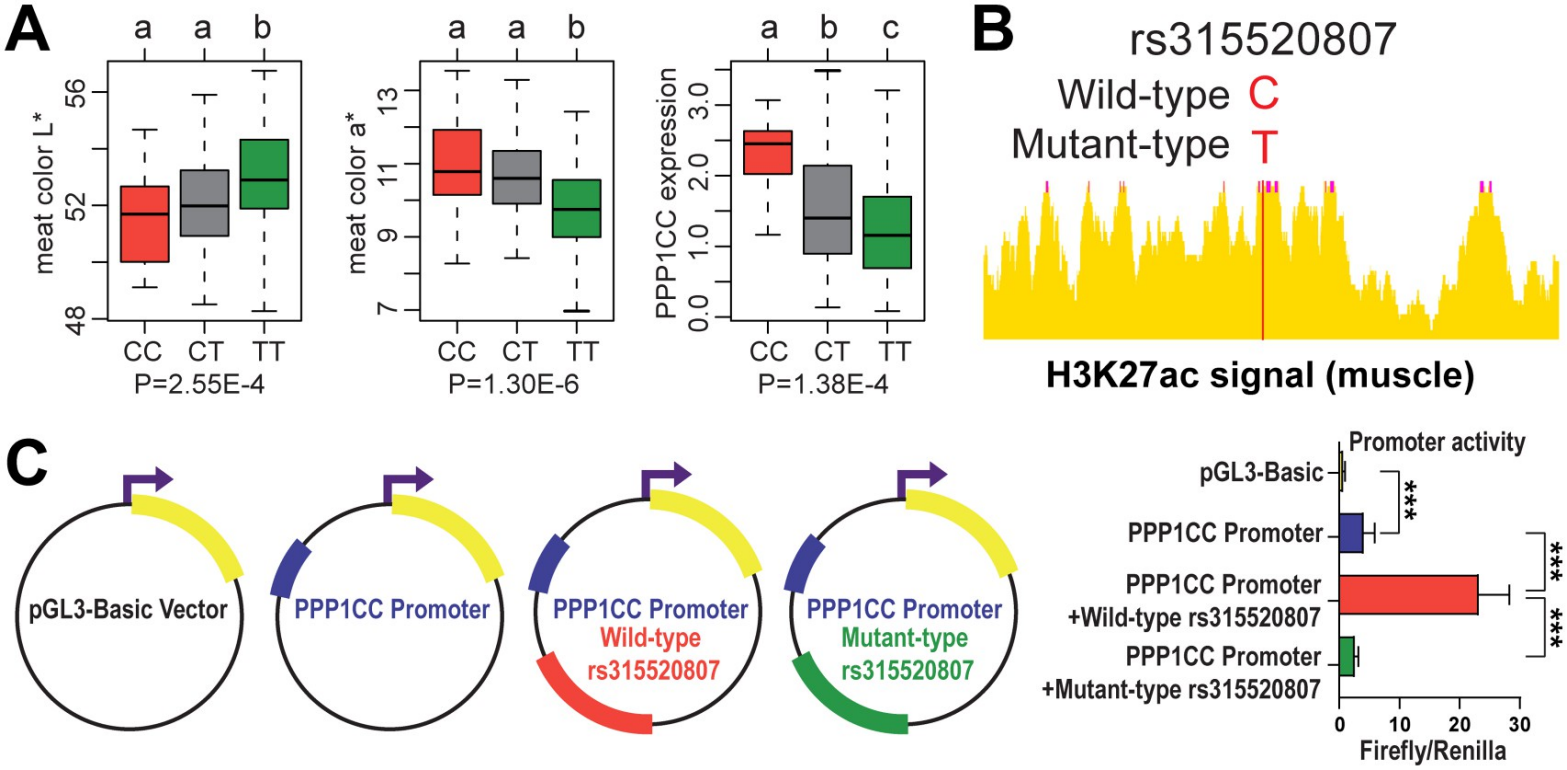
Screening and functional analysis of potential causative mutation. (A) The genetic effects of the SNP rs315520807 (GGA15: 6298343) on meat color L*15 min, a*15 min and *PPP1CC* expression. The indicated p values are based on one-way ANOVA. Box plots indicate the median (centerline), 25^th^-75th percentiles (limits), and minimum and maximum values (whiskers). (B) The rs315520807 variant located in H3K27ac signal peak detected in chicken muscle. (C) Enhancer effects of the wild-type rs315520807 fragment and the mutant fragment on *PPP1CC* promoter activity. The empty pGL3-basic vector was used as a control. The purple arrow represents the transcription site. The blue frame represents the *PPP1CC* promoter, and the yellow frame represents the luciferase reporter gene. The red and green frames represent the wild-type rs315520807 fragment and the mutant-type fragment, respectively. For all tests, triplicate performed and used to calculate the mean and standard deviation (SD). *** *P* < 0.001.

The SNP rs315520807 (GGA15: 6298343) was the most significant SNP in GWAS for meat color and explained 3.33% and 3.53% of the phenotypic variances in L*15 min and a*15 min, respectively. Individuals with the mutant-type TT exhibited significantly higher L* value and lower a* value than those with the wild-type CC. The *PPP1CC* expression levels in breast muscle among the three genotyping groups were significantly different (Fig 5A). Based on genome-wide regulatory elements atlas in chicken muscle [22], the rs315520807 variant located in H3K27ac signal peak (Fig 5B).

Considering that rs315520807 located in the intron of *PPP1CC*, we tested its enhancer effect on the promoter of *PPP1CC* in the pGL3-basic vector. First, we ligated the promoter fragment of *PPP1CC* into the pGL3-basic vector. Then, we ligated the wild-type (WT) rs315520807 fragment and the mutant rs315520807 fragment to the pGL3 vector containing the *PPP1CC* promoter. We co-transfected HeLa cells with each of the four vectors together with the pRL-TK Renilla luciferase plasmid, measured their luciferase activities and calculated the ratio. Compared with that of the vector carrying only the *PPP1CC* promoter, the luciferase activity of the vector carrying both the WT rs315520807 fragment and the *PPP1CC* promoter was significantly increased by 5.74 times (*P* = 5.00E-04). However, when the fragment was replaced with mutant-type rs315520807, the luciferase activity was reduced to be consistent with that of the vector carrying only the *PPP1CC* promoter. The results revealed that the fragment where rs315520807 is located might be an enhancer that activates the *PPP1CC* promoter, and the potential causative mutation rs315520807 (C > T) nullified its enhancer effect (Fig 5C). This finding was consistent with the observed differences in the gene expression of *PPP1CC* among different alleles *in vivo*.

### Divergence between two different chicken breeds

The fast-growing white-feathered chickens and local chickens used in this study were distinct chicken breeds. The divergence of meat color phenotypes, myoglobin content, frequency of the variant rs315520807, expression of *PPP1CC* and fast-twitch fiber marker genes were compared between these two breeds.

**Fig 6:**
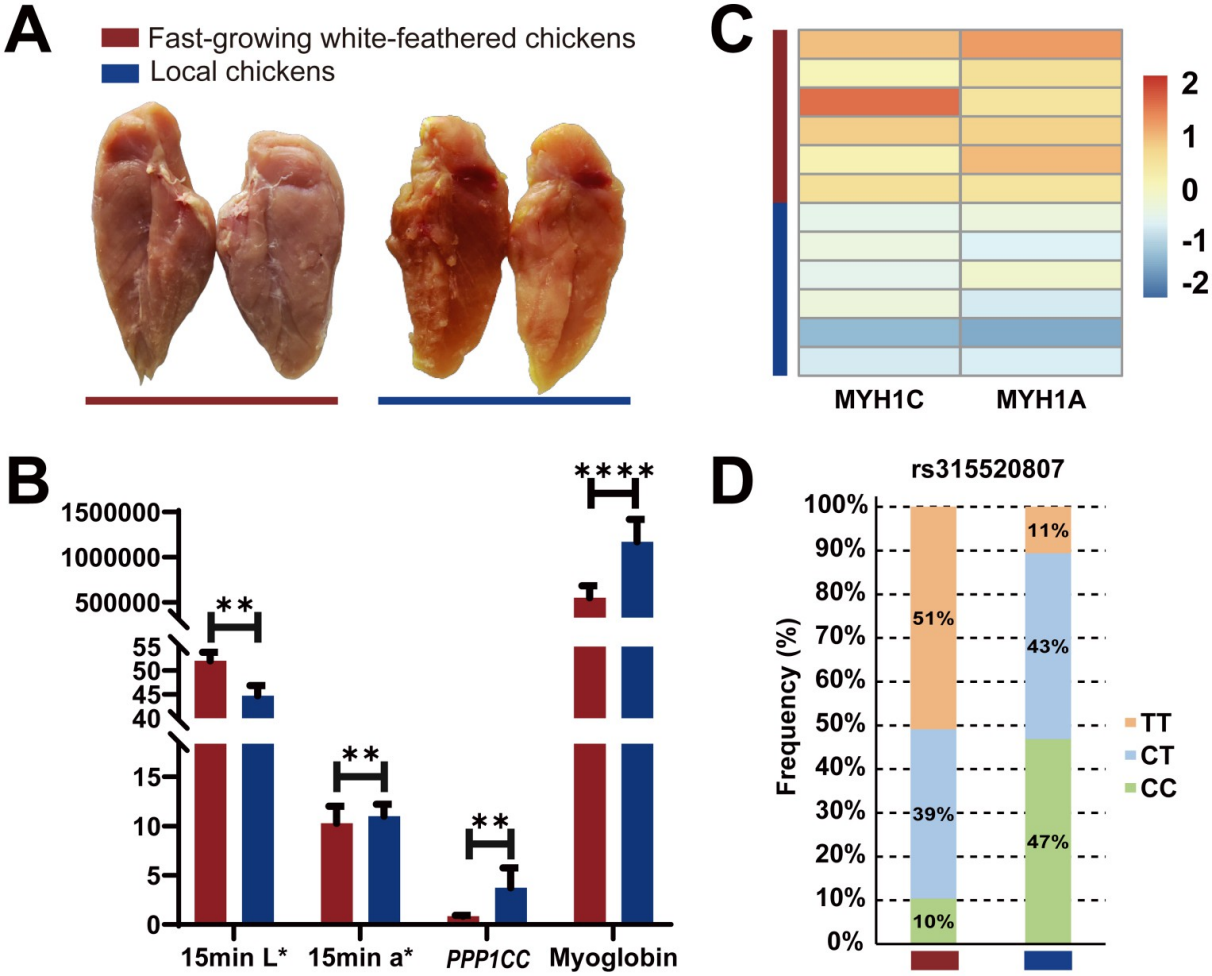
Comparisons between fast-growing white-feathered chickens and local chickens. (A) Pictures to show the breast muscle color of fast-growing white-feathered (left) and local (right) chickens. (B) Results of L*15 min, a*15 min, mRNA expression of *PPP1CC* and myoglobin content of breast muscles from two different breeds. (C) Results of the expression of fast-twitch muscle fiber marker genes. The bottom and top sides of the color bar represent the minimum and maximum values, respectively. (D) The allele frequency distributions of rs315520807 in 230 fast-growing white-feathered chickens and 80 local chickens.

Compared with those of local chickens, the breast muscles of fast-growing white-feathered chickens presented a significant increase (*P* < 0.01) in L*15 min and a notable decrease (*P* < 0.01) in a*15 min (Fig 6A and 6B). The myoglobin content in the breast muscles of fast-growing white-feathered chickens was significantly lower (*P* < 0.0001) than that in local chickens (Figs 6B and S7).

In addition, the expression of *PPP1CC* was significantly lower (*P* < 0.01) and that of the fast-type muscle fiber genes *MYH1A* and *MYH1C* were significantly greater (|fold change| > 1.5, *P* < 0.05) in the breast muscle of white-feathered chickens than in that of local chickens (Fig 6B and 6C). Similarly, the frequency of the rs315520807 TT allele in fast-growing white-feathered chickens (51%) was significantly higher than that in local chickens (11%) (Fig 6D).

## Discussion

In this study, we assessed meat color using the L* and a* phenotypes. We performed a GWAS by using 230 individuals and more than nine million SNPs to calculate a genome-wide distribution via genome resequencing, and the results revealed that only one significant signal was observed on chromosome 15, which might be associated with chicken meat color. The gene-based association analysis, which associations at the gene level by aggregating genotypes for each gene [17], indicated that only *PPP1CC* at the loci was significantly associated with the meat color L* and a* values.

To confirm the causal relationships, MR analysis was used to provide more solid evidence for causal inference between *PPP1CC* expression and target traits. The results revealed that *PPP1CC* has negative effects on meat color L* and positive effects on a*. Additionally, the findings suggested effects on meat color traits beyond *PPP1CC* gene expression, emphasizing the need for further study in the future.

The *PPP1CC* is indispensable for muscle glycogen metabolism and functions by binding with protein phosphatase 1 regulatory subunit 3A (*PPP1R3A)*. In chickens, *PPP1R3A* has been identified as the functional gene for the pH of the pectoralis major muscle in pH-divergent selected lines [23]. In our study, the expression of *PPP1R3A* decreased in *PPP1CC*-SSKO mice (fold change = 0.75, *P* = 0.01). Previous studies on PP1 have focused on its regulatory subunits and shown that disruption of gene expression causes weight gain, insulin resistance, and fat deposition [24]. The clarification of the role of *PPP1CC* in meat color is reported here for the first time.

For a gene such as *PPP1CC* with a broad function and a phenotype such as meat quality that is affected by post-slaughter metabolism, skeletal muscle-specific knockouts that are time inducible are essential. Given that PP1γ has 99.38% percent identity between mice (PP1G_MOUSE) and chickens (Q5ZL39_CHICK), we generated a mouse model in which the expression of the target gene was reduced to 13.35% of the original expression two weeks after induction. Significant changes in the meat lightness and myoglobin content in the limb muscles of this model were detected, without significant differences in meat redness.

Further comparison via metabolomic and transcriptomic analyses suggested that consistent differences in gene expression and metabolites related to glycolysis occurred. We found that the levels of four key metabolites increased significantly in *PPP1CC*-SSKO mice, which indicated the presence of more glycolytic substrates with greater potential for conversion to lactate and H^+^ after slaughter, allowing muscle acidification. Previously, the protein kinase AMP-activated noncatalytic subunit gamma 3, *PRKAG3* [25], and *PHKG1* [11] genes were identified as causative genes for glycogen content in skeletal muscle in pigs. The abnormal expression of *PRKAG3* in pigs and mice leads to increased glycolytic potential and acidic meat [26]. The abnormal expression of *PHKG1* in pigs can also lead to an increase in glycolytic potential and acidic meat [11]. Interestingly, the three genes identified as causative genes for meat quality were all related to the glycogen metabolism network.

Meat quality is related to myofiber type [27]. In particular, differences were also found in the myofiber-type specification pathway genes, including *ATP2A2* and *PPARGC1A*, in *PPP1CC*-SSKO mice. Knockdown of *ATP2A2* by short interfering RNA (siRNA) repressed MHC-β/slow expression [28]. *PPARGC1A*, an important slow-twitch myofiber mediator, was decreased (fold change = 0.54, *P* = 7.6E-4), as was its cooperator *NFATC2* (fold change = 0.58, *P* = 1.0E-3), which indicated that the generation of slow-twitch myofibers was inhibited in *PPP1CC*-SSKO mice. The fast-twitch myofiber ratio increased, and biomarkers of fast-twitch myofiber, including *TNNI2*, *TNNT3*, and *TNNC2*, tended to increase. The well-known feature of fast-twitch myofibers is enhanced glycolysis, which is consistent with the above results. Additionally, the fact that *PPP1CC* was grouped with fast-twitch and slow-twitch myofiber marker genes in a single network indicates that *PPP1CC* might be connected to myofiber-type specification.

We also provided experimental evidence verifying the effect of the potential causative variant rs315520807 (C > T) on *PPP1CC* expression. The fragment containing the wild-type CC (rs315520807) might be the enhancer that activates the promoter of *PPP1CC*, and the mutant-type TT results in the loss of the enhancer effect. We also predicted the motifs in the fragment containing the rs315520807 variant and annotated the transcription factor binding sites. A motif containing the rs315520807 variant was predicted as the binding site for KLF9 (S8 Fig), and KLF9 had multiple binding sites in the promoter of PPP1CC (S7 Table). The regulation of *PPP1CC* expression by rs315520807 through its interaction with the transcription factor KLF9 requires further validation.

The breeding objective for fast-growing white-feathered chicken breeds primarily emphasizes achieving elevated breast muscle weight, a criterion markedly distinct from that of local breeds. In our investigation, compared with that of local breeds, the L*15 min value was notably greater, and the a*15 min value was lower in fast-growing breeds, as previously reported [29]. The pigment primarily responsible for a*15 min, myoglobin, exhibited reduced levels in fast-growing chicken breeds. This discrepancy in meat color is also consistent with the increased frequency of the potential causative variant rs315520807 mutant type TT (51% vs 11%) and the decreased expression of *PPP1CC* in fast-growing chicken breeds, which may also be related to variations in muscle fiber types, as evidenced by the heightened expression of type II marker genes in fast-growing breeds. The frequency of the rs315520807 wild type CC in the fast-growing chickens is 10%, which can serve as an effective genetic marker in breeding programs for improving meat color and reducing the incidence of PSE-like meat.

There are several limitations of the current study. The downregulation of *PPP1CC* was related to the lightness change 15 min after slaughter, but the corresponding lightness changes observed in *PPP1CC*-SSKO mice were recorded 24 h after slaughter. This discrepancy might be related to the species differences because fast-twitch myofibers, which are the main component of the chicken pectoralis major muscle, undergo fast glycolysis after slaughter, whereas slow-twitch myofibers, which are the main component of mouse muscle, have a slow glycolysis rate. The rate of glycolysis after slaughter differed between chickens and mice. Additionally, L*15 min and L*24 h as well as a*15 min and a*24 h are highly phenotypically correlated in the chicken population studied (S8 Table). It remains uncertain whether rs315520807 is the causative mutation due to the interference of linkage disequilibrium among sites, and further investigation is needed to determine that.

## Conclusions

We found that *PPP1CC* expression was causally related to breast muscle lightness and redness in chickens using large-scale genome-wide association, gene-based association analysis and Mendelian randomization analysis. Using *PPP1CC*-SSKO mice, we also found that lightness increased and myoglobin content decreased in the limb muscle, and the mechanisms by which *PPP1CC* influences meat color included myofiber-type specification. Functional validation through a plasmid reporter assay revealed that the SNP rs315520807 (C > T) located in the intron of *PPP1CC* could regulate gene transcription activity. The differences in the meat color phenotypes and related indices between fast-growing white-feathered chickens and local chickens support the conclusions above. Our results identified *PPP1CC* as the causative gene for meat color and identified a novel target gene and variant for the innovation of meat improvement technology.

## Materials and methods

### Ethics statement

The study protocol was approved by the Ethics Review Committee of the Institute of Animal Sciences (IAS) of the Chinese Academy of Agricultural Sciences (CAAS) (reference no. IAS2023-3) and was conducted in strict accordance with the Regulations for the Administration of Affairs Concerning Experimental Animals established by the Chinese Ministry of Science and Technology.

### Experimental birds

The fast-growing white-feathered chickens were produced and raised by Foshan Gaoming Xinguang Agricultural and Animal Industrials Co., Ltd. (Foshan, China). The 230 fast-growing white-feathered chickens (101 males and 129 females) were randomly selected and slaughtered at 42 days of age.

The fast-growing white-feathered chickens and Beijing-you chickens were raised under the same conditions at the Institute of Animal Sciences (IAS), Chinese Academy of Agricultural Sciences (CAAS) until they reached 42 days of age. Ten breast muscle samples from each breed were subjected to myoglobin analysis. Additionally, six breast muscle samples from each breed were subjected to transcriptome analysis. Details of the chicken breeds analyzed across different studies are provided in S9 Table.

### Experimental mice

The mice used in the study had a C57BL/6 background. The *PPP1CC*-SSKO deletion, *PPP1CC*^(fl/fl; ACTA1-creER(Tg))^, was generated by mating the actin alpha 1, skeletal muscle-Cre 1 (ACTA1) mouse line with the *PPP1CC*^flox/flox^ mouse line. The B6.Cg-Tg(ACTA1-cre/Esr1*)2Kesr/J mouse line was provided by The Jackson Laboratory [30]. The *PPP1CC*^flox/flox^ mouse line was generated by Biocytogen Pharmaceuticals Co., Ltd. (Beijing, China) by inserting LoxP sites flanking exon 4 to exon 6 of the *PPP1CC* gene and was maintained at our facilities as an inbred strain. All *PPP1CC*-SSKO mice had a C57BL/6 background, and each experimental animal was genotyped for homozygous floxed alleles and positive heterozygosis Cre alleles. Age- and sex-matched *PPP1CC*^(fl/fl;ACTA1-creER(0))^ littermates were used as controls for *PPP1CC*-SSKO mice. The mice were kept on a daily 12 h light/dark schedule, given access to tap water *ad libitum*, and fed a standard chow diet (Research Diets, D12450J). The total number of 9-week-old mice in this study was 20, including 10 controls and 10 *PPP1CC*-SSKO mice.

### Genotyping and quality control

Blood (1.5 mL) was collected from each chicken using the wing vein method, placed in an anticoagulant tube containing EDTA-K2, and stored at -20°C after mixing. Genomic DNA was extracted from blood samples using the phenol-chloroform method.

Whole-genome sequencing (WGS) of 230 fast-growing, white-feathered chickens was conducted on the Illumina NovaSeq 6000 platform, with an average depth of coverage of approximately 10× [31]. Variant calling was performed according to a standardized bioinformatics pipeline [32,33]. Specifically, trimmed sequencing data were aligned to the chicken reference genome (GRCg6a/galGal6) using the Burrows–Wheeler Aligner (BWA)-MEM algorithm [34]. Then, PCR duplicates were removed with Picardtools v1.1152. Variant calling was performed via HaplotypeCaller in GVCF mode with joint genotyping of all samples. We used ANNOVAR software [35] and the existing genome annotation file (gff) to annotate each detected SNP. The SNPs were filtered with the GATK Variant Filtration protocol. The filtering settings were as follows: variant confidence score < 30.0, QualByDepth < 2.0, ReadPosRankSum < −8.0, total depth of coverage < 4.0, and FisherStrand > 60.0. In addition, quality control was conducted using the following criteria: individual call rate ≥ 90%, SNP call rate ≥ 90%, and minor allele frequency (MAF) ≥ 0.05. After filtering, 9,760,228 autosomal variants in the 230 fast-growing, white-feathered chickens remained.

In addition, we downloaded whole genome sequencing data for Beijing-you chickens (n = 80) with accession numbers CRA004519 from GSA database [36] for the allele frequency collection.

### Phenotype collection

Phenotypes were collected for all chickens and mice. The meat color (L*, a*, b*) of the chicken pectoralis major muscle was measured using the CIELAB color space system (Konica Minolta, CR-410) [37], which consists of opponent-color scales based on the opponent-color theory of human color vision, where a* indicates redness when positive and greenness when negative, b* indicates yellowness when positive and blueness when negative, and L* (lightness) describes the relationship between reflected and absorbed light, with a value of 100 for white and 0 for black. The CIELAB color space system (Konica Minolta, CR-400) was used to measure the color of the hindlimb muscles of mice. The color of the meat for each mouse is shown in S10 Table. Traits related to muscle fiber characteristics were obtained using the myofibrillar NADH staining method (pH = 7.4) on quadriceps from 9-week-old control (n = 4, female) and *PPP1CC*-SSKO (n = 3, female) mice. The quantification of type I and type II muscle fibers was conducted through manual microscopic analysis. Three regions were selected, and 100 cells within each region were identified on each slide.

### RNA extraction and quantitative real-time PCR

Hindlimbs from 9-week-old control (n = 5) and *PPP1CC*-SSKO (n = 5) mice were collected. Total RNA was isolated using TRIzol reagent (Invitrogen) according to the manufacturer’s protocol, and cDNA was synthesized using a RevertAid First Strand cDNA synthesis kit (Thermo). Quantitative real-time PCR was performed in duplicate using SYBR Green PCR Master Mix on a real-time PCR instrument (ABI 7500 detection system, Applied Biosystems) with a reaction volume of 20 μL. All primer sequences are listed in S11 Table. All PCR samples were quantitated using the comparative CT method to obtain relative quantifications that were normalized to 18S rDNA.

### Genome-wide association study

L*15 min, a*15 min, and *PPP1CC* mRNA expression data were used as phenotypes for the GWAS. The GWAS was performed using a univariate linear mixed model (LMM) implemented in GEMMA (version 0.98.1) [38]. Sex was considered a covariate for all traits. The univariate LMM was expressed by the following equation:

$$y=W\alpha +x\beta +u+\varepsilon ;u∼MVN_{n}(0,\lambda \tau^{-1}K),\varepsilon ∼MVN_{n}(0,\tau^{-1}I_{n})$$

where **y** represents a 230-vector of quantitative traits for 230 individuals; **W** represents a 230⊆2 matrix of intercepts (column with ones) and sex (0 for males and 1 for females); **α** represents a 2-vector of the corresponding coefficients, including the intercept; **x** represents a 230-vector of marker genotypes; **β** represents the effect size of the markers; **u** represents a 230-vector of random effects, which is estimating genetic relationships among individuals from all SNPs; **ε** represents a 230-vector of errors; τ^−1^ represents the variance of the residual errors; λ represents the ratio between the two variance components; K is a known 230×230 relatedness matrix; **I**_n_ is a 230×230 identity matrix; MVN_*n*_ represents the 230-dimensional multivariate normal distribution; and n is 230.

The Wald test was used as a criterion to select the SNPs associated with the phenotypes. The whole-genome threshold was corrected by the Bonferroni correction (0.05/9,760,228), and the suggestive significance threshold was 1/9,760,228. Manhattan and quantile–quantile (Q–Q) plots were constructed for each trait using the CMplot package (https://cran.r-project.org/web/packages/CMplot/index.html) in R (version 4.2.1). The genes in the genome-wide significant and suggestive regions were identified using the UCSC annotation of the GRCg6a/galGal6 genome.

We identified the loci linked with the most significant SNP within 500 kb by performing pairwise r^2^ measurements using the package LDBlockShow [39].

### Gene-based association analysis

To identify candidate genes for meat color from the GWAS data, we performed a gene-level association analysis using GWAS summary statistics in MAGMA (v1.10) [40]. We first applied the chicken genome annotation information (GRCg7b) and the combined genotypes to generate the gene annotation file. Next, we leveraged the GWAS summary statistics to perform the gene-level association analysis. Finally, we set *P* < 1.00E-06 as the significance threshold to filter the candidate genes in the gene-level association.

### Mendelian randomization analysis

The GWAS summary statistics for L*15 min, a*15 min, and *PPP1CC* mRNA expression were used for MR analysis. To fulfill the three assumptions for instrument variables [41], genetic variants that were significantly associated with the expression of *PPP1CC* (*P* < 5.41E-9) but not with a*15 min and L*15 min were screened. After clustering and removing high-LD sites (r^2^ > 0.9), 20 SNPs were selected as instrumental variables for *PPP1CC* expression. *PPP1CC* expression was defined as the exposure trait, and a*15 min and L*15 min were the outcome traits. This MR model is described in S9 Fig.

We performed MR analysis using five different methods, including inverse-variance weighting (IVW) [42,43], MR–Egger regression [44,45], weighted median [46], and mode-based estimation (MBE) [47] using simple mode and weighted mode, implemented in the “TwoSampleMR” R package for robust validation. *P* > 0.05 in MR–Egger regression and *P* < 0.05 in the other four methods were considered valid. A consistent effect across the five methods is less likely to be a false positive. In this study, sensitivity analyses consisted of heterogeneity and horizontal pleiotropy tests. The MR–Egger and IVW methods were used to test for heterogeneity, and MR–Egger was used to test for horizontal pleiotropy. These sensitivity analysis results are presented in S3 Table.

### Tamoxifen induction and genotype identification

To induce robust Cre activity in mouse skeletal muscle, we injected tamoxifen at 6 weeks of age for 5 consecutive days. The procedure was performed according to the protocol of the Jackson Laboratory [48]. Briefly, tamoxifen was administered via intraperitoneal injection once every 24 hours for 5 consecutive days. The determined injection dose by weight was approximately 0.075 mg tamoxifen/g body weight.

Tamoxifen (Solarbio Life Science, cat. no. IT0030) was diluted with corn oil (Solarbio Life Science, cat. no. C7030). We monitored the mice for any unusual signs or soreness during injection. The mice only experienced weight reduction throughout the injection period, which is consistent with the statistical results of the Jackson Laboratory. For genotype identification, genomic DNA extracted from mouse tails was subjected to PCR using primers specific for 5’loxP, 3’loxP, and Cre (S11 Table). The PCR conditions were as follows: 95°C for 3 min; 32 cycles at 94°C for 30 s, 60°C for 30 s, and 72°C for 30 s; and 72°C for 5 min. For 5’loxP, the product size of the WT allele was 471 bp, and the product size of the mutant allele was 589 bp. For 3’loxP, the product size of the WT allele was 294 bp, and the product size of the mutant allele was 383 bp. For Cre, the product size of the transgene was 248 bp, and the product size of the internal positive control was 521 bp (S10 Fig).

### Western blot analysis

Total cell lysates were obtained from the quadriceps of mice through lysis using RIPA lysis buffer (Beyotime, China) supplemented with 1% PMSF, followed by homogenization using a vortex oscillator (Roche, USA). Protein concentrations were determined using the Enhanced BCA Protein Assay Kit (Beyotime, China). An equal volume of 5X loading buffer was subsequently added to the samples, which were then subjected to a 10-minute boiling step. The proteins were separated by 10% SDS–PAGE, transferred onto a 0.45 mm PVDF membrane, and subsequently blocked for 2 hours at room temperature with 5% non-fat dry milk. The membrane was then incubated overnight at 4°C with the appropriate primary antibody. Next, the membrane was incubated with the secondary antibody for an additional hour. The developed membranes were exposed to SuperSignal West Pico PLUS Chemiluminescent Substrate (Thermo, USA). Notably, the primary antibodies against PPP1CC (SAB5700201) were obtained from Sigma–Aldrich (Merck KGaA, Darmstadt, Germany), and those against α-tubulin (AB4074) were procured from Abcam (Cambridge, UK).

### Myoglobin determination

The triceps brachii muscles of 10 control and 10 9-week-old *PPP1CC*-SSKO mice and the breasts of 8 fast-growing, white-feathered chickens and Beijing-you chickens were used for myoglobin (MYG MOUSE, MYG CHICK) determination. The meat was homogenized in lysis buffer (100 mM Tris-HCl pH 8.5, 7 M urea, 1% SDS, 5 mM TCEP, and protease inhibitor cocktail) at room temperature. The protein concentration was determined using a bicinchoninic acid (BCA) assay. Fifty micrograms of protein was reduced with 5 mM TCEP at 56°C for 30 min and alkylated with 20 mM iodoacetamide at room temperature for 30 min in the dark. The proteins were subsequently filtered with a 10 kDa ultrafiltration device and washed three times with 50 mM TEAB buffer (pH 8.0). The protein obtained from ultrafiltration was subsequently resuspended in 100 μl of digestion buffer composed of 50 mm TEAB buffer. Then, trypsin was added at a ratio of 1:25 (w/w), and protein digestion was performed overnight at 37°C. The peptide was washed twice using ultrafiltration buffer with 1% formic acid and dried using a SpeedVac. Finally, the peptide was resuspended in 0.1% formic acid and 2% acetonitrile for subsequent nano-LC–MS/MS analysis.

Nano-LC–MS/MS analysis was performed using an Orbitrap Fusion Tribrid MS (Thermo Scientific, San Jose, CA) equipped with a nanospray flex ion source coupled with a Dionex UltiMate 3000 RSLC nanosystem (Thermo, Sunnyvale, CA). Peptide samples (2 μL) were injected into the PepMap C18 columns (75 μm × 3 mm, 3 μm) at 6 μL/min for online enrichment and then separated on a PepMap C18 column (2 μm, 75 μm × 250 mm) with 0.1% formic acid as buffer A and 0.1% formic acid in 80% acetonitrile as buffer B at 300 nL/min. The peptides were eluted with the following gradient: 0–5 min, 2% B; 5–38 min, 2%–22% B; 38–45 min, 22–99% B; 45–50 min, 99% B; 50–51 min, 98–2% B; and 51–60 min, 2% B.

The mass spectrometers were operated using electrospray ionization (2 kV) at 275°C in “top speed” mode. The Orbitrap resolution was 120,000, and for tandem mass spectrometry (MS/MS), the Orbitrap resolution was 30,000. The MS/MS spectra were acquired using a quadrupole isolation width of 1.2 m/z and HCD normalized collision energy (NCE) of 30. Hemoglobin quantitative peptide tandem mass spectrometry was shown in S7 Fig.

### Differentially expressed gene analysis

Six breast muscle samples from white-feathered chickens and Beijing-you chickens were randomly selected for transcriptome sequencing. Hindlimbs from 9-week-old control mice (n = 5, male) and *PPP1CC*-SSKO mice (n = 5, male) were collected, and RNA samples were isolated. Male mice were chosen for this analysis because they presented a more significant difference in L*24 h than female mice did. RNA sequencing was conducted by Annoroad Gene Technology Co., Ltd. (Beijing, China). The reference genome for mice was *Mus musculus* GRCm38.90, and the reference genome for chickens was GRCg6a (GCA_000002315.5).

The criteria for significantly differentially expressed genes were |fold change| > 1.5 and *P* < 0.05. Kyoto Encyclopedia of Genes and Genomes and Gene Ontology analyses were conducted for both chickens and mice as previously described [29]. The 903 genes that were differentially expressed between *PPP1CC*-SSKO mice and control mice at 9 weeks of age are shown in S12 Table. The gene expression data of the chickens is shown in S13 Table.

Differentially expressed genes in mice related to both muscle fiber specification and slow- and fast-fiber markers were uploaded into STRING [49] (https://cn.string-db.org/) bioinformatics software with default parameters and all interaction sources.

### Targeted metabolomics analysis and metabolite identification

Metabolite extraction and metabolomics analysis were performed on quadriceps of six control mice and six *PPP1CC*-SSKO mice that were 9 weeks of age using mass spectrometry (MS). The tissue sample was placed in 700 μl of 80% v/v HPLC-grade methanol that had been chilled to -80°C for two hours (70 mg). A superfine homogenizer was used to grind the mixture on dry ice. The mixture was then vortexed three times for ten seconds each, incubated at 80°C for eight hours, and centrifuged at 12000 rpm at 4°C for 20 minutes. The sample supernatant was transferred to a fresh tube (1.5 ml Eppendorf), where it was concentrated using a vacuum system (Thermo Scientific Savant Vac) until it was dry (4 hours). For the redissolution of residues for additional analysis, 80% methanol was used.

Targeted metabolomic analysis was implemented using TSQ Quantiva (Thermo Fisher Scientific). Reverse-phase chromatography (C18 column) with 10 mM tributylamine and 15 mM acetate in water as mobile phase A and 100% methanol as mobile phase B was performed. The TCA cycle, glycolysis pathway, and pentose phosphate pathway were the focus of this analysis. In this experiment, we used a 25-min gradient from 5% to 90% for mobile phase B. Data collection was performed by switching between positive and negative ions. Both Q1 and Q3 had a resolving power of 0.7 full width at half maximum (FWHM). The positive and negative ion source voltages were 3,500 V and 2,500 V, respectively. The source parameters were as follows: heater temperature, 300°C; auxiliary gas flow rate, 10; sheath gas flow rate, 35; spray voltage, 3,000 V; and capillary temperature, 320°C. Metabolite identification was performed using a custom-built database and TraceFinder 3.2 (Thermo Fisher Scientific) as described previously in [50].

The threshold for significantly differentially abundant metabolites was |fold change| > 1.5, orthogonal partial least squares discriminant analysis (OPLS-DA) score > 1, and *P* < 0.05. OPLS-DA (S11 Fig) was conducted using MetaboAnalyst 5.0 (https://www.metaboanalyst.ca/).

### Luciferase reporter assay

The promoter fragment of *PPP1CC* (1500 bp upstream of the transcription start site) was synthesized, cloned, and inserted into the pGL3-basic vector. The wild-type rs315520807 and the mutant-type rs315520807 fragments (150 bp upstream and downstream) were synthesized, cloned, and inserted into the pGL3 vector containing the *PPP1CC* promoter. The synthetic sequences of candidate regions were provided in S14 Table. HeLa cells were plated at a density of 1.9 × 10^5^ per well in 24-well plates 1 day before transfection and were cultured under adherent conditions in high-glucose DMEM (HyClone) + 10% fetal bovine serum (FBS, Gibco). The cells were transfected with 475 ng (per well) of pGL3 plasmids containing different segments of the *PPP1CC* gene sequence and 25 ng (per well) of the pRL-TK Renilla luciferase plasmid using Lipofectamine 8000 (Beyotime). Luciferase activities were determined 36 h after transfection using the Dual-Luciferase Reporter Assay System (Promega) according to the manufacturer’s instructions. Luciferase bioluminescence measurements were performed with a Tecan Infinite 200 Pro. All of the experiments were conducted in triplicate, and the firefly luciferase activity was normalized to the Renilla luciferase activity of each sample.

To investigate whether the mutant-type rs315520807 were located within transcriptional regulatory motifs, the MEME suite was used [51]. Subsequently, a detailed in silico annotation of the identified motifs was conducted by the JASPAR program with their default settings [52].

### Statistical analysis

Statistical analysis was carried out with SPSS 25.0 software (SPSS, Inc., Chicago, IL). The Student’s two-tailed t-test was used for phenotypes and qPCR. One-way analysis of variance (ANOVA) followed by Duncan’s multiple range test were used for multiple group comparisons in haplotype analysis. The bar charts depict the results as the means ± standard errors of the means, which were visually represented using GraphPad Prism 8.0 software. *P* < 0.05 was considered significant for the Student’s two-tailed t-test, one-way ANOVA and Duncan’s multiple range test. The sample numbers for each analysis are indicated in the figure legends.

## Supporting information

S1 Fig The QQ plot of GWAS for L*15 min and a*15 min of fast-growing white-feathered chickens. (TIF)

S2 Fig The gene-based association analysis using the GWAS summary statistics for meat color L*15 min and a*15 min by MAGMA. Each dot represents a gene. The dotted line indicated the whole-genome significance threshold (*P* < 0.05/11,821 = 4.23E-06).
(TIF)

S3 Fig Leave-one-out sensitivity test and pleiotropic effects of MR analysis. Leave-one-out sensitivity test: MR leave-one-out sensitivity analysis for *PPP1CC* at a*15 min (A) and L*15 min (B). Circles indicate MR estimates for *PPP1CC* on meat color using the inverse-variance weighted fixed-effect method if the SNP was omitted. The bars indicate the CIs of the MR estimates. Funnel plot of the MR analysis for a*15 min (C) and L*15 min (D). The funnel plot was used to evaluate the presence of possible heterogeneity across the estimates, which indicates potential pleiotropic effects. The figure presents the observed causal effect of each of the 20 instrumental variables (IVs) as dots and the average causal effect of all IVs combined (β IV) using the inverse variance weighted (solid line) and MR–Egger (dashed line) methods on the x-axis. The Y axis presents the inverse standard error of the estimated causal effect for each of the single-nucleotide polymorphisms (IVs).
(TIF)

S4 Fig Representative results of NADH staining in mouse hindlimb muscle from a mouse. Dark purple indicates slow-twitch myofibers; light purple indicates fast-twitch myofibers; scale bar = 500 μm.
(TIF)

S5 Fig The GO enrichment of differentially expressed genes related to muscle fiber composition in the hindlimb muscle of 9-week-old mice. Differentially expressed genes were selected based on a |fold change| > 1.5 and *P* < 0.05. The GO biological processes were related mainly to muscle function (“muscle system process”, “regulation of response to stimulus”, “muscle contraction”, and “regulation of muscle system process”), cytoskeleton (“contractile fiber”, “myofibril”, “contractile fiber part”, “myosin filament”, “myosin complex”, “sarcomere”, “intercalated disc”) and muscle fiber activity (“actin binding”, “cytoskeletal protein binding”, “actin filament binding”, and “microfilament motor activity”).
(TIF)

S6 Fig Annotation of differentially expressed genes involved in the glycolysis pathway in the hindlimb muscle of 9-week-old mice. Differentially expressed genes with a |fold change| > 1.5 were selected. The key gene involved in the dehydrogenation reaction, glyceraldehyde-3-phosphate dehydrogenase (GADPH), was upregulated, and the key gene involved in the hydrogen depletion reaction, lactate dehydrogenase (LDH), was downregulated in *PPP1CC*-SSKO mice; the key rate-limiting enzymes hexokinase (HK) and phosphofructokinase (PFK) were upregulated. The red box represents upregulated genes, and the green box represents downregulated genes.
(TIFF)

S7 Fig Hemoglobin quantitative peptide tandem mass spectrometry. Three specific peptides with high mass spectrometry signals and stable enzymatic cleavage products were selected for targeted quantitative analysis by analyzing the peptide sequences of trypsin-digested hemoglobin.
(TIF)

S8 Fig Prediction of transcription factors binding before and after mutation of rs315520807. The dark red boxes represent coding exons. Predicted KLF9 binding sites containing the variant rs315520807 were presented in the box.
(TIF)

S9 Fig Flow chart of Mendelian randomization analysis. (TIF)

S10 Fig Agarose gel electrophoresis for identification of mouse genotypes. (TIF)

S11 Fig Orthogonal partial least squares discriminant analysis (OPLS-DA) of targeted metabolomics data from the hindlimb muscles of 9-week-old mice. (TIF)

S1 Table Genome-wide significant SNPs for meat L*15 min and a*15 min. (XLSX)

S2 Table Effects of *PPP1CC* on L*15 min and a*15 min using different MR methods. (XLSX)

S3 Table Heterogeneity and pleiotropy tests for exposure to outcome. (XLSX)

S4 Table Differentially expressed genes and GO biological process terms related to muscle function. (XLSX)

S5 Table Changes in myofiber specification gene expression in *PPP1CC*-SSKO mice compared to control mice. (XLSX)

S6 Table Details of significantly differentially abundant metabolites (*PPP1CC*-SSKO vs control). (XLSX)

S7 Table The multiple binding sites of KLF9 in the promoter of PPP1CC. (XLSX)

S8 Table Estimates of genetic parameters correlations and phenotypic correlations between meat color traits in fast-growing white-feathered broilers. (XLSX)

S9 Table Information of chickens used across different analyses. (XLSX)

S10 Table Meat color phenotypes of mice at 9 weeks of age. (XLSX)

S11 Table Primers used for reverse transcription and real-time PCR. (XLSX)

S12 Table Differentially expressed genes between *PPP1CC-SSKO* mice and control mice at 9 weeks of age. (XLSX)

S13 Table Differentially expressed genes between white-feathered fast-growing chickens and Beijing-you chickens at 42 days of age. (XLSX)

S14 Table The synthetic sequence of PPP1CC promoter, wild-type rs315520807 fragment and mutant-type rs315520807 fragment. (XLSX)
