# Supplementary figures and images for: Protein phosphatase 1 catalytic subunit gamma is a causative gene for meat lightness and redness
Source: PLoS Genet. 2024 Nov 20;20(11):e1011467. doi: 10.1371/journal.pgen.1011467 (PMC11616877; doi:10.1371/journal.pgen.1011467)

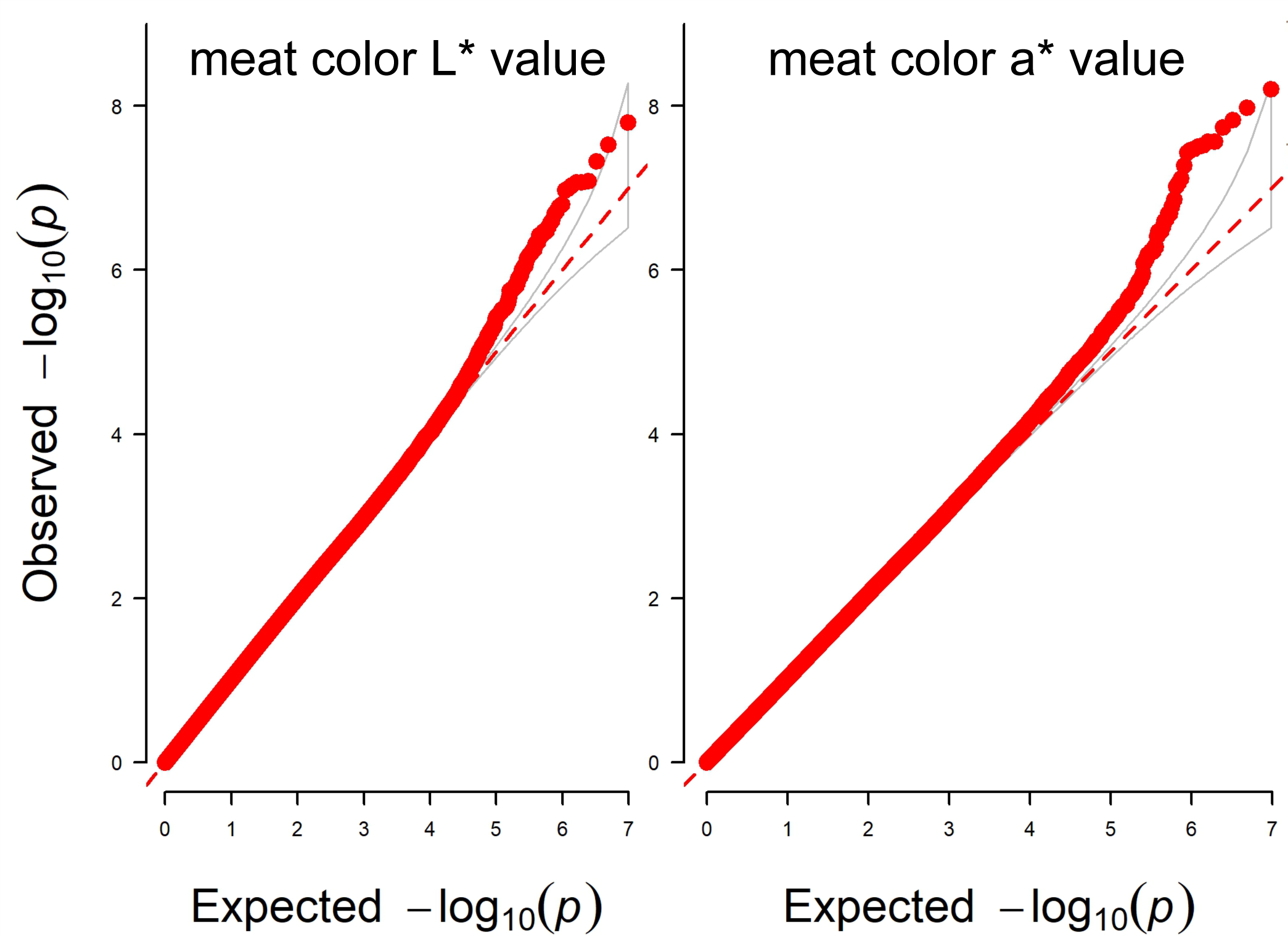

Supplement: S1 Fig — (TIF) [file pgen.1011467.s001.tif]

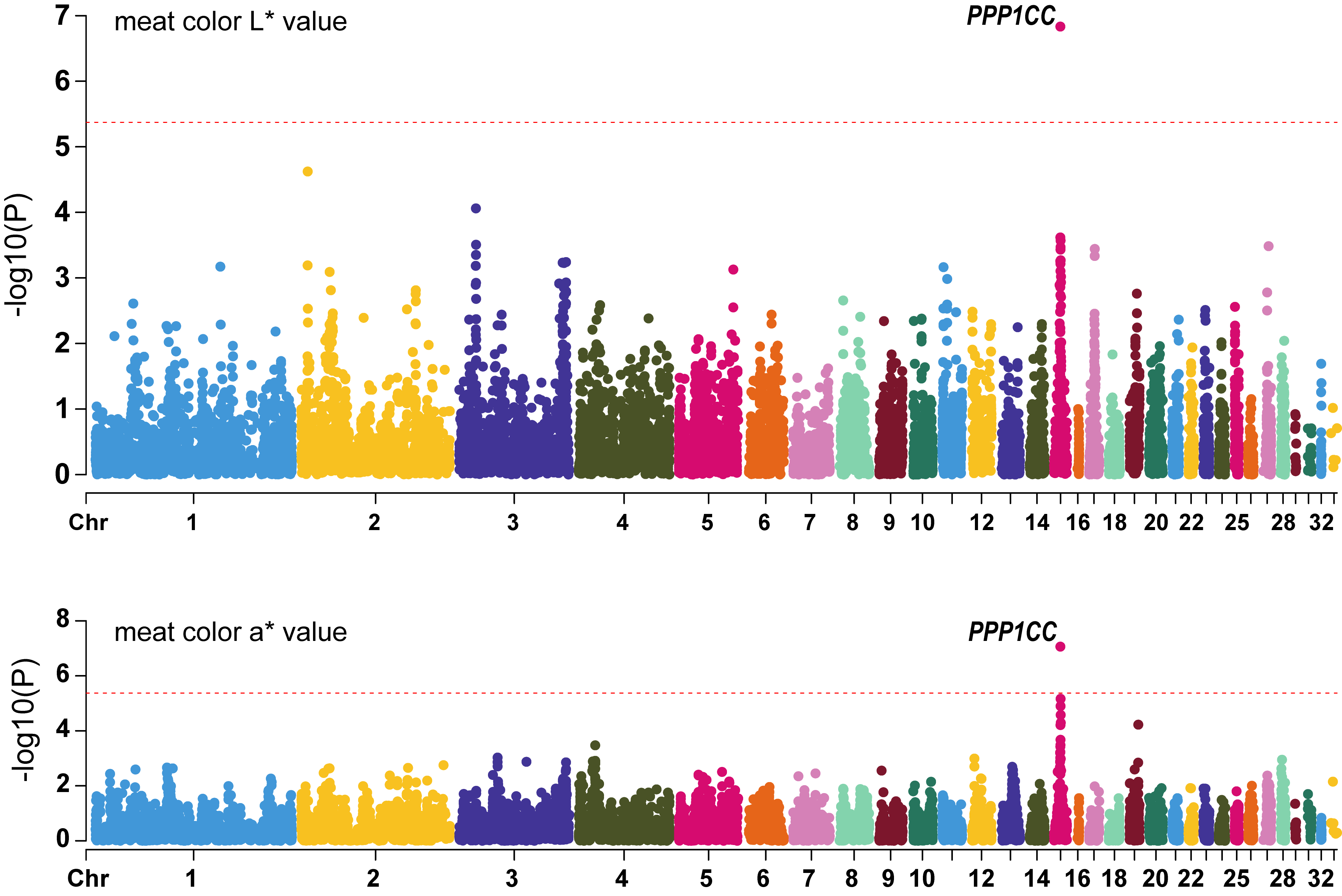

Supplement: S2 Fig — Each dot represents a gene. The dotted line indicated the whole-genome significance threshold (P < 0.05/11,821 = 4.23E-06). (TIF) [file pgen.1011467.s002.tif]

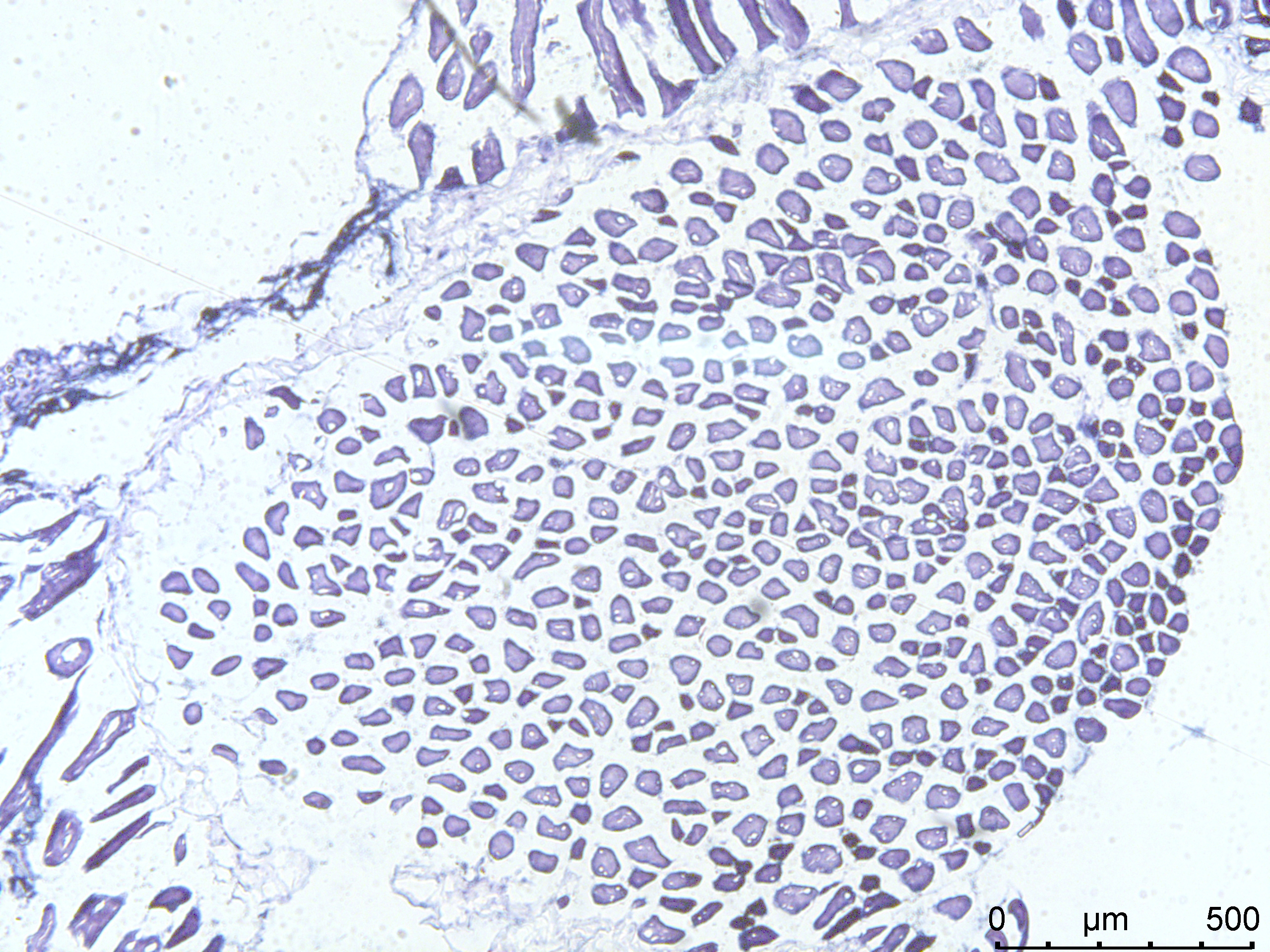

Supplement: S4 Fig — Dark purple indicates slow-twitch myofibers; light purple indicates fast-twitch myofibers; scale bar = 500 μm. (TIF) [file pgen.1011467.s004.tif]

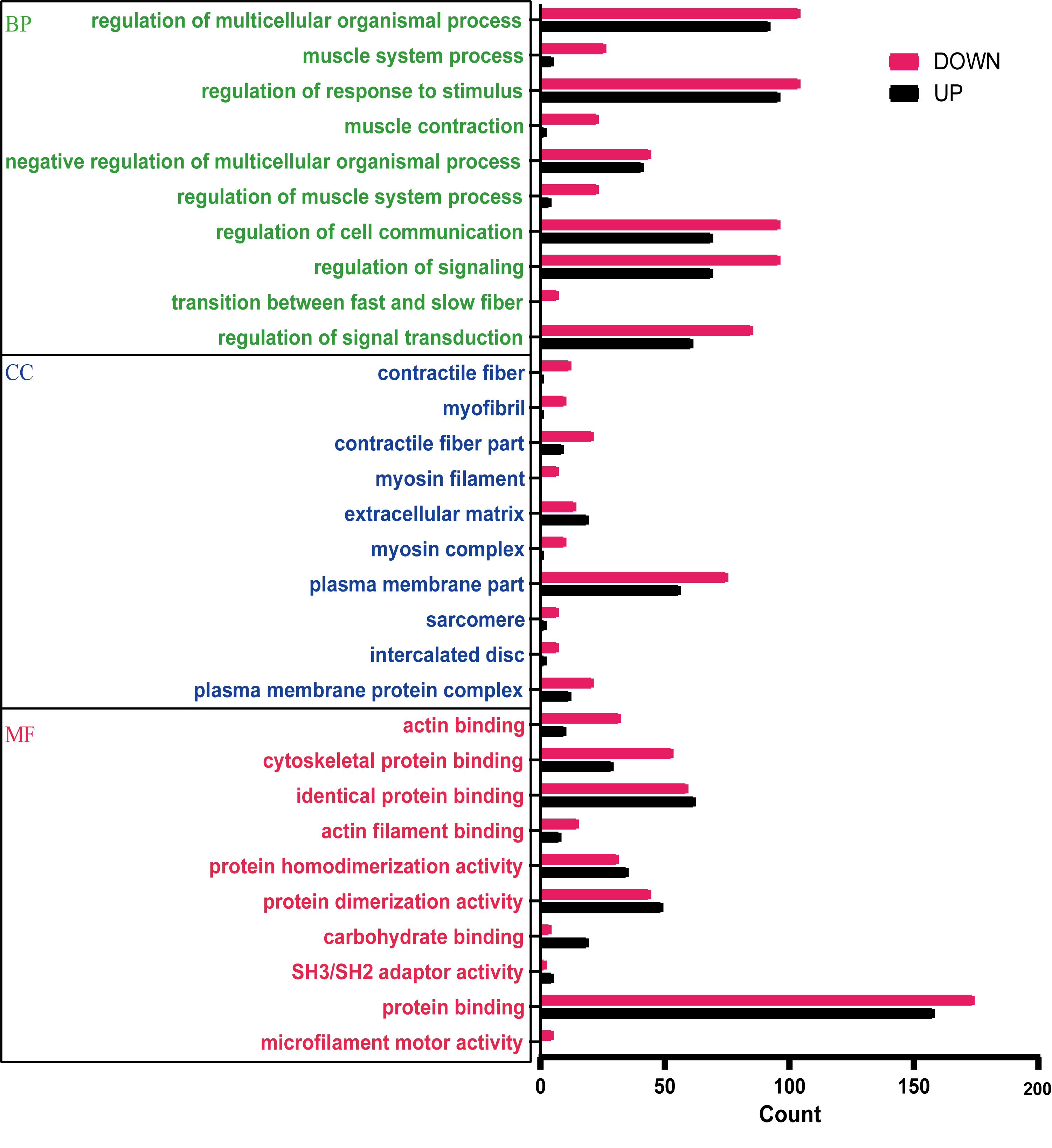

Supplement: S5 Fig — Differentially expressed genes were selected based on a |fold change| > 1.5 and P < 0.05. The GO biological processes were related mainly to muscle function (“muscle system process”, “regulation of response to stimulus”, “muscle contraction”, and “regulation of muscle system process”), cytoskeleton (“contractile fiber”, “myofibril”, “contractile fiber part”, “myosin filament”, “myosin complex”, “sarcomere”, “intercalated disc”) and muscle fiber activity (“actin binding”, “cytoskeletal protein binding”, “actin filament binding”, and “microfilament motor activity”). (TIF) [file pgen.1011467.s005.tif]

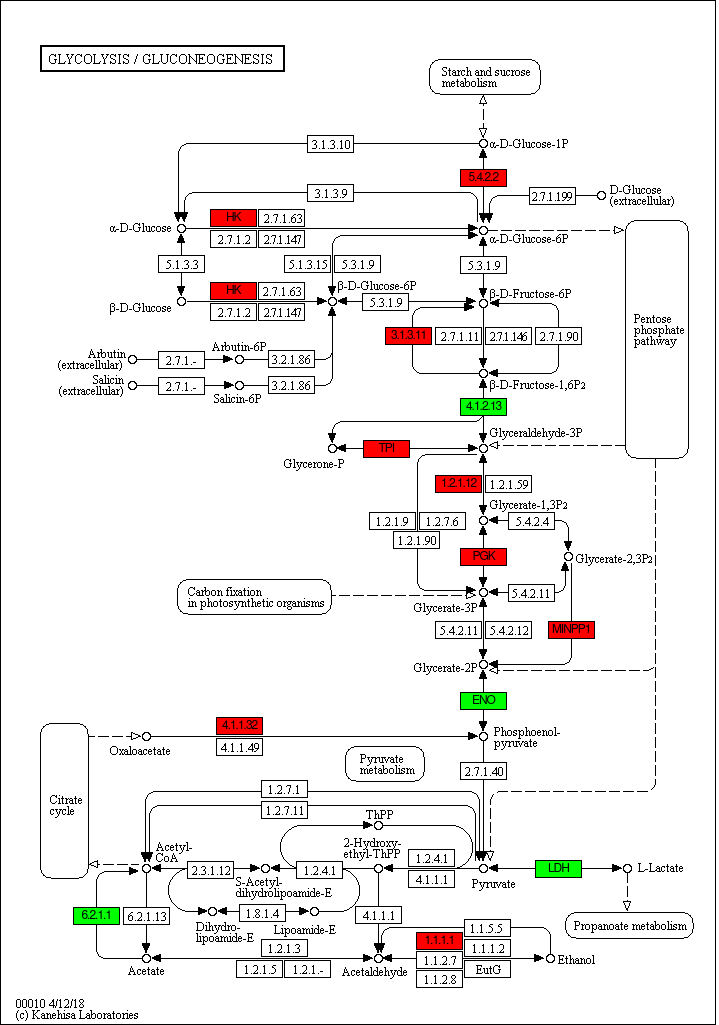

Supplement: S6 Fig — Differentially expressed genes with a |fold change| > 1.5 were selected. The key gene involved in the dehydrogenation reaction, glyceraldehyde-3-phosphate dehydrogenase (GADPH), was upregulated, and the key gene involved in the hydrogen depletion reaction, lactate dehydrogenase (LDH), was downregulated in PPP1CC-SSKO mice; the key rate-limiting enzymes hexokinase (HK) and phosphofructokinase (PFK) were upregulated. The red box represents upregulated genes, and the green box represents downregulated genes. (TIFF) [file pgen.1011467.s006.tiff]

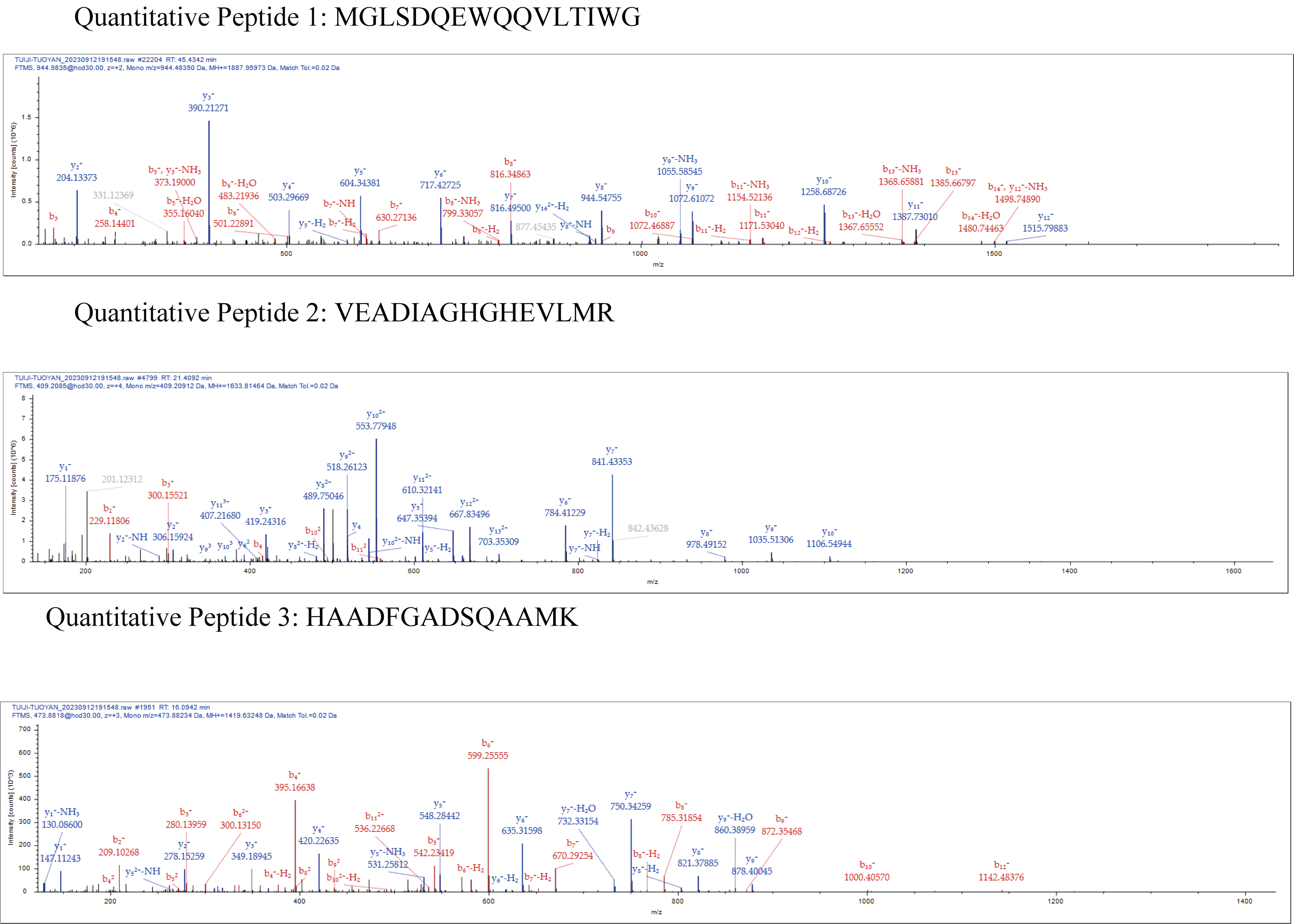

Supplement: S7 Fig — Three specific peptides with high mass spectrometry signals and stable enzymatic cleavage products were selected for targeted quantitative analysis by analyzing the peptide sequences of trypsin-digested hemoglobin. (TIF) [file pgen.1011467.s007.tif]

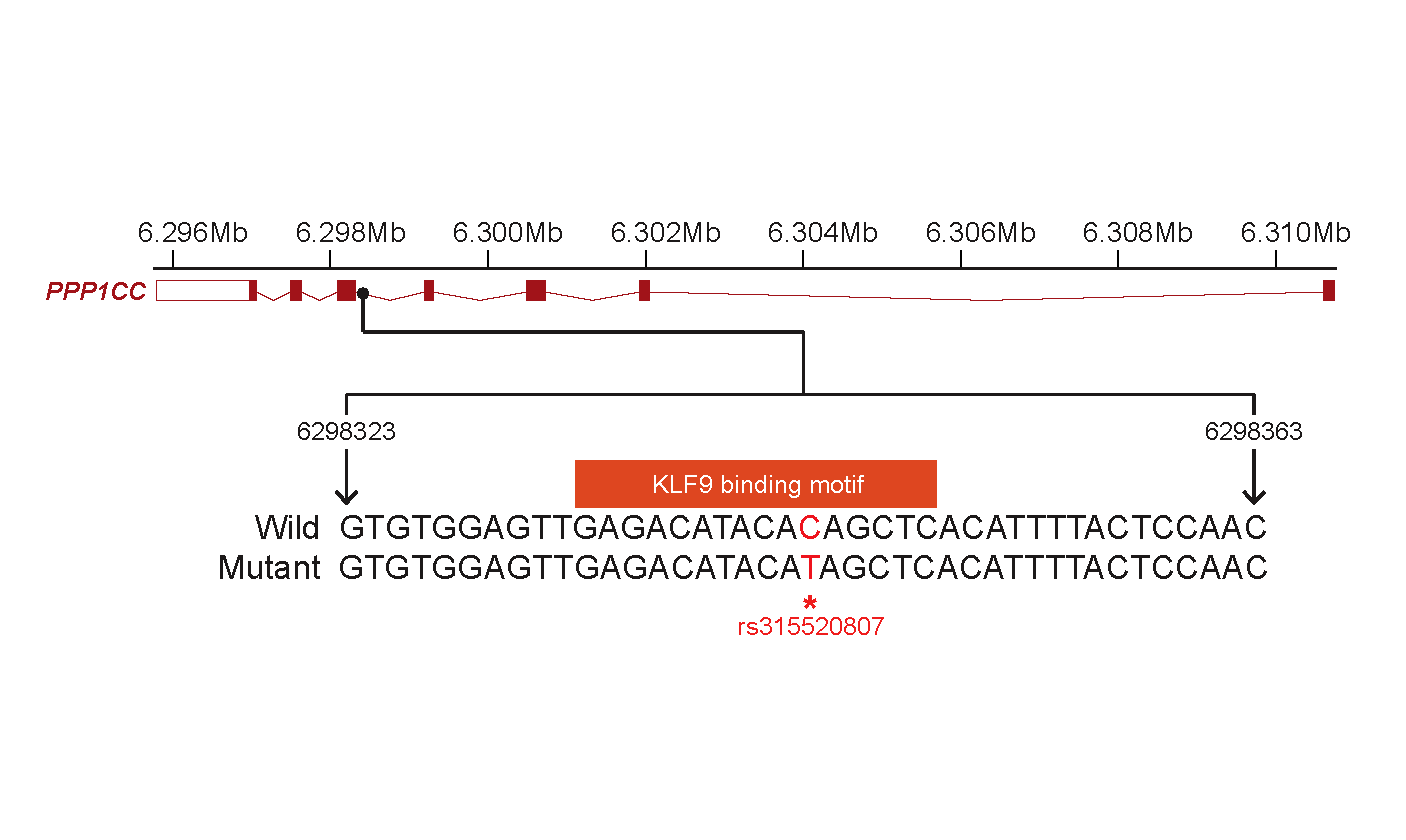

Supplement: S8 Fig — The dark red boxes represent coding exons. Predicted KLF9 binding sites containing the variant rs315520807 were presented in the box. (TIF) [file pgen.1011467.s008.tif]

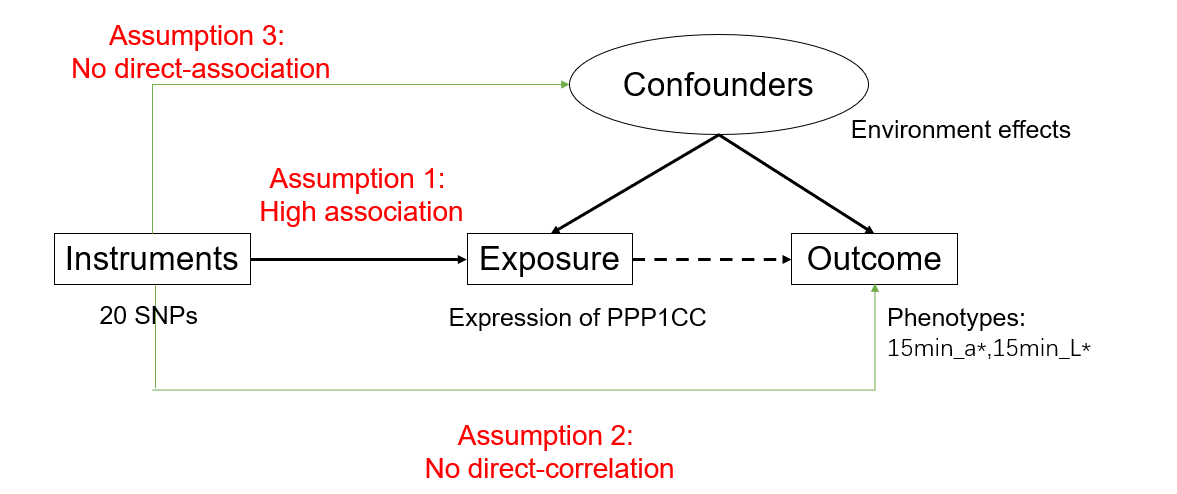

Supplement: S9 Fig — (TIF) [file pgen.1011467.s009.tif]

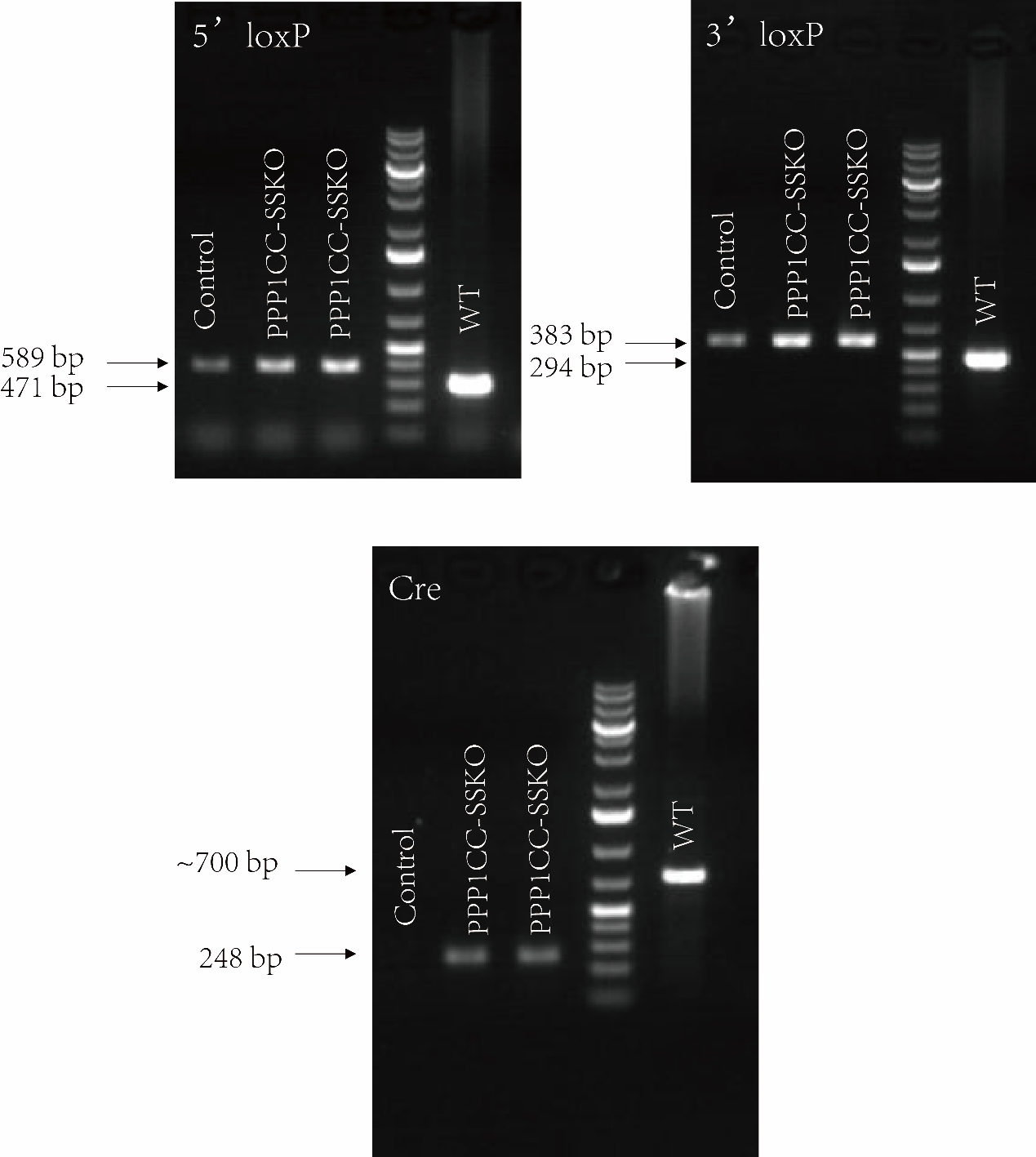

Supplement: S10 Fig — (TIF) [file pgen.1011467.s010.tif]

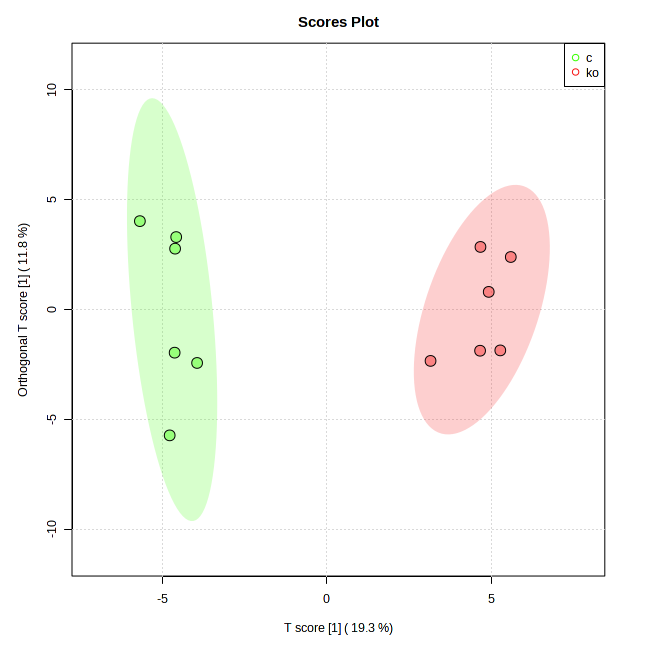

Supplement: S11 Fig — (TIF) [file pgen.1011467.s011.tif]
